# Probing High-Order Transient Oligomers Using Ion Mobility Mass Spectrometry Coupled with Infrared Action Spectroscopy

**DOI:** 10.1021/acs.analchem.4c02749

**Published:** 2024-08-16

**Authors:** Sjors Bakels, Steven Daly, Berk Doğan, Melissa Baerenfaenger, Jan Commandeur, Anouk M. Rijs

**Affiliations:** † Division of Bioanalytical Chemistry, Department of Chemistry and Pharmaceutical Sciences, Amsterdam Institute of Molecular and Life Sciences, Vrije Universiteit Amsterdam, De Boelelaan 1105, 1081 HV Amsterdam, The Netherlands; ‡ Centre for Analytical Sciences Amsterdam, 1098 XH Amsterdam, The Netherlands; § MS Vision, Spectrometry Vision B.V., Televisieweg 40, Almere 1322 AM, The Netherlands

## Abstract

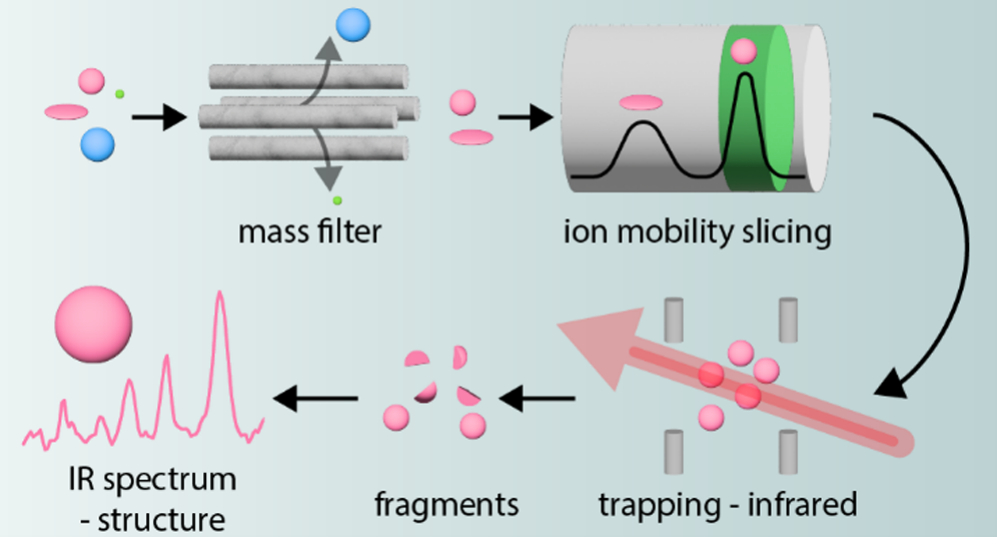

Understanding and controlling peptide aggregation are critical due to its neurotoxic implications. However, structural information about the key intermediates, the oligomers, is obscured by a cascade of coinciding events occurring at various time and energy scales, which results in complex and heterogeneous mixtures of oligomers. To address this challenge, we have developed the Photo-Synapt, a novel, multidimensional spectrometer that integrates ion mobility mass spectrometry with infrared (IR) action spectroscopy within a single experiment. By combining three different orthogonal analytical dimensions, we can select and isolate individual oligomers by mass, charge, size, and shape and provide a unique molecular fingerprint for each oligomer. The broad application of this technology is demonstrated by its application to oligosaccharide analysis from glycoproteins, which are challenging to analyze due to the minute differences between isomers. By integration of IR action spectroscopy with ion mobility mass spectrometry, this approach adds an analytical dimension that effectively addresses this limitation, offering a unique molecular fingerprint for each isomer.

Nature has many examples where not only biological function, but also malfunction arises from complex processes. An important example of such a highly complex molecular mechanism is that of aggregation of peptides and proteins. Aggregation, the transition from soluble functioning proteins into insoluble amyloid aggregates, is directly related to several human neurodegenerative diseases including Alzheimer’s and Parkinson’s disease.^1−7^ In these aggregation processes, complexity arises from the diversity of intermediate soluble oligomeric species and the numerous possible reaction pathways that connect them. Our understanding of the early stages of protein aggregation is hampered by the heterogeneity that results. This is particularly important as neurotoxicity originates from the soluble, oligomeric intermediates that are formed early on the aggregation pathway.^2,3^ Understanding this complexity and unraveling the early steps of the molecular aggregation network are key to regulate and ideally stop these phenomena. This requires an experimental approach that can selectively pick one specific intermediate from the heterogeneous ensemble at specific points in time and unravel its structure.

Ion mobility-mass spectrometry (IM-MS) has proven to be a powerful approach in elucidating structural information on specific ions within a heterogeneous mixture of low abundant analytes.^8^ Here, the mobility of ions is measured, allowing for the separation of these ions based on their size, mass, shape, and charge. IM-MS has been employed in studying the aggregation process, probing the formation of early stage aggregation intermediates.^7,9−19^

Using IM-MS, oligomers of different sizes but with the same *m*/*z* values can be separated. In such experiments, mass spectrometry is used to select a specific set of oligomers with the same *n*^*z*+^ ([*n*M + *z*H]^*z*+^) where *n* is the number of monomer units in the oligomer and *z* is the number of charges. Subsequently, IM is used to separate individual oligomers with the same *m*/*z* ratio, and to identify each oligomer by their mobility and mobility selected mass spectra.^18,19^ Moreover, their overall structure can be deduced via their collision cross sections.^7,20^ However, IM-MS is not sensitive to the secondary structure and so cannot directly measure the expected increase in β-sheet character upon oligomer formation. To be able to directly measure such secondary structural changes, mass- and mobility-selected infrared (IR) spectroscopy measurements are required. For example, Bowers, von Helden and Pagel et al. have combined IR spectroscopy with IM-MS using a home-built setup to probe the secondary structure of the segments NFGAIL from the unstructured human islet amyloid polypeptide (hIAPP, diabetes type II) and the peptide VEALYL from the insulin B chain. Using the C=O stretching vibrations (amide I band) between 1600 and 1800 cm^–1^, they could probe the onset of β-sheet formation.^14,21^

The above-mentioned example is part of a growing trend of combining ion mobility mass spectrometry with either IR action spectroscopy and/or UV photodissociation (UVPD),^22−29^ either based on fully home-build instruments or modified commercial ion-mobility mass spectrometers, which has been reviewed in detail by Stroganova and Rijs.^30^ Although the home-built systems have the benefit of being fully flexible, they lack the ease of use and support base of commercially available mass spectrometers. Moreover, these home-built spectrometers are typically integrated with free electron lasers at facilities and/or cryogenic-cooled IR spectroscopic methods.^31,32^ Focusing on the commercially based mass spectrometers, two types of hyphenated instruments exist; those starting with IR-MS where the ion mobility options are added later, or ion mobility mass spectrometers where optical access is integrated. For the first option, either differential mobility spectrometry (DMS)^33^ or field asymmetric ion mobility spectrometry (FAIMS)^34^ has been placed at the front end of the mass spectrometer. Both DMS and FAIMS provide extra separation capabilities to the existing IR-MS workflow and are easy to implement. The high electric field nature of FAIMS, structural information via collision cross-section determination is not possible. In recent years, modifying commercial MS instruments to add the spectroscopy capability has become more popular, by for example providing optical access and optical elements to direct the laser beam predominantly adding IR and/or UVPD to trapping mass spectrometers. Although only limited examples are shown for IM-MS with IR spectroscopy, UVPD (and XUV^35^) has been demonstrated previously on a Synapt,^22,23^ making it possible to probe isomeric species or conformational changes by UVPD after IM separation. These experiments could be performed in the trap or transfer TWave, where the pressures are ∼5 × 10^–2^ mbar and 8 × 10^–3^ mbar, respectively. For IR spectroscopy, pressures of <10^–5^ mbar are required with flexible irradiation times, demanding a different design.

Therefore, we have developed the Photo-Synapt, an ion mobility mass spectrometer (IM-MS) interfaced with IR action spectroscopy in a single experiment. This was achieved by customizing a Synapt G2 mass spectrometer (Waters) to facilitate optical access, ion trapping, and ion mobility slicing. An extra vacuum chamber was incorporated between the transfer TWave and the TOF region. This new chamber facilitates differential pumping to ensure the low pressure required for IR ion spectroscopy. Moreover, it contains two hexapoles and pin traps,^36,37^ which can be used to trap and manipulate ions over a long time. Optical access was arranged both orthogonally in the second pin trap and parallel with the ion propagation direction. Tunable IR light is provided using a small, turn-key table-top laser. Ion trapping and manipulation are performed using pulsed voltages controlled by a custom-made software. This includes modification of the existing ion optics to allow for ion mobility slicing to be performed.

The Photo-Synapt spectrometer, its modifications, and mode of operations are described in detail in the following section, while its wide scientific capabilities are demonstrated on selected experiments. Here, we present the application of using the Photo-Synapt to study the early steps of peptide aggregation. Using mass-selection, a series of oligomers with the same *m*/*z* can be selected, and subsequently, a single oligomer can be physically isolated via ion mobility slicing. We measured oligomer-specific vibrational spectra via IR action spectroscopy. Additionally, the broad impact of this three-dimensional IM-MS-IR combination has shown structural identification of the sialic acid linkage isomers in glycans from glycoproteins. With the Photo-Synapt, it becomes possible to acquire unique mass- and ion-mobility-selected IR spectra from mixtures of α2,6- and α2,3-linked sialic acid isomers, enabling their individual analysis and characterization.

Combining ion mobility, mass spectrometry, and IR spectroscopy in one experiment offers significant potential as a powerful analytical tool, allowing us to analyze the overall shape and size while obtaining detailed structural insights from vibrational spectroscopy. This hyphenated approach, integrating three orthogonal dimensions, facilitates the reduction of conformer heterogeneity and complexity and enables the separation and structural identification of molecular isomers and oligomers.

## Modifications of the Synapt

**Figure 1:**
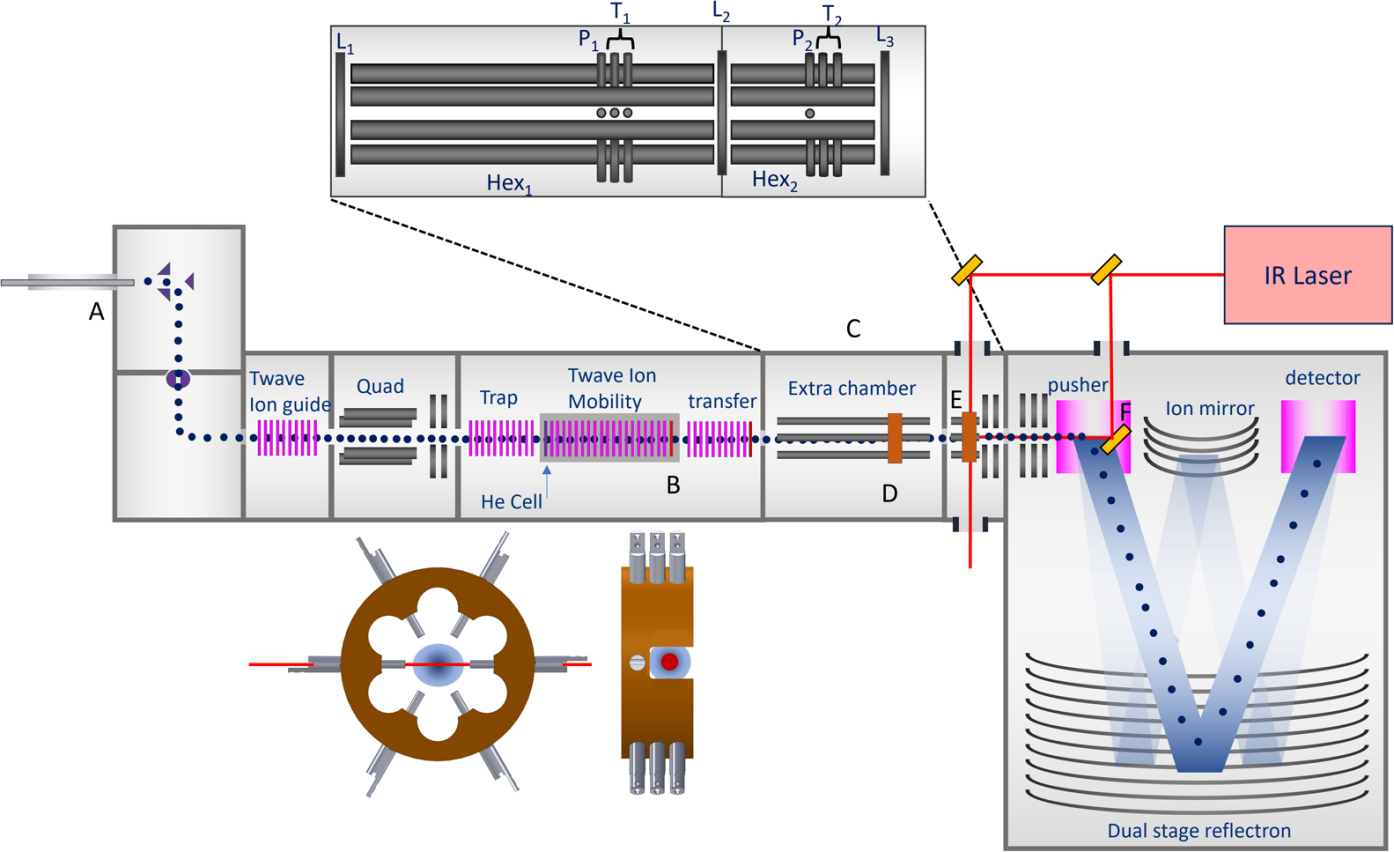
A schematic representation of the Photo-Synapt detailing the modifications indicated by the capital letters. (A) Pressure sleeve, (B) ion mobility slicing, (C) additional vacuum chamber. (D) First hexapole and pin trap, (E) second hexapole and pin trap, (F) gold mirror for on-axis irradiation. Optical access is also possible perpendicularly to irradiate ions in the second pin trap. An expanded view of the additional ion optics for ion trapping is shown in the top inset, see text and the Supporting Information for further details. The bottom inset shows a front and side view of the pin traps, indicating the position of the trapped ions and the perpendicular irradiation scheme.

Figure 1 shows a schematic of the Photo-Synapt with its modifications to allow ion mobility slicing, ion trapping, and UV/IR photofragmentation spectroscopy to be combined in a single experiment. The voltages presented in this section are for using the Photo-Synapt in positive mode.

### Source

Unmodified electrospray (ESI) and static nanospray (nano-ESI) sources were used to study the glycoproteins and VEALYL oligomers, respectively. To obtain better collisional cooling and hence improved transmission of high *m*/*z* species, the TWave ion guide was modified by inclusion of a pressure sleeve, which causes an increase in the pressure within the ion guide.^36,38^ In addition, a valve was added to the vacuum line of the source chamber, which can be closed to further increase the pressure. The combined measures increase desolvation and transmission of large ions, and the modifications are presented in Figure S1 of the Supporting Information.

### Ion Mobility Slicing

**Figure 2:**
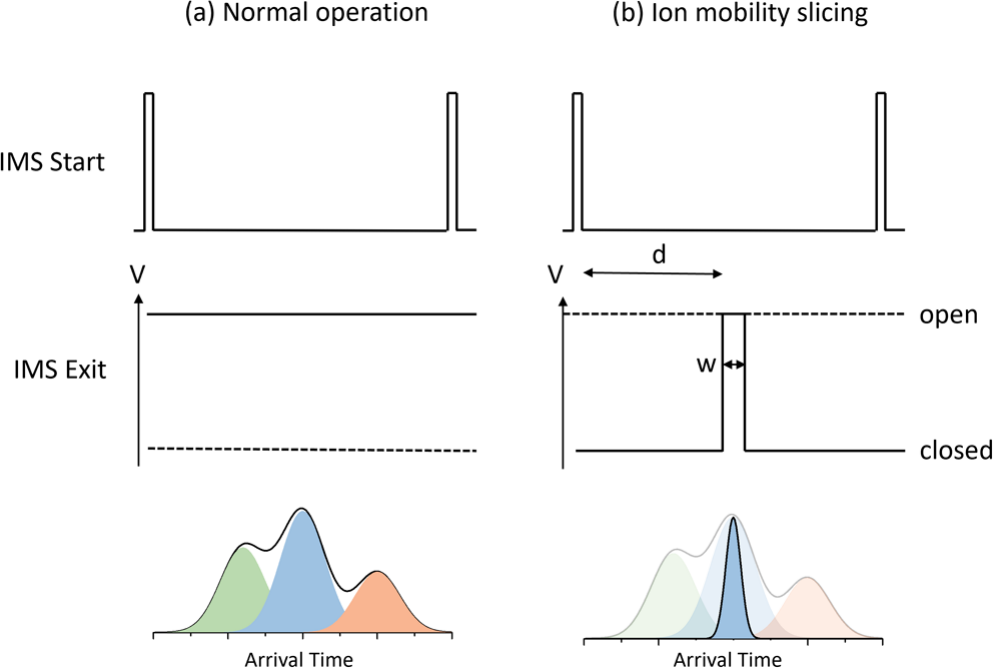
Schematic representation of ion mobility slicing in the Photo-Synapt: (a) during normal operation, the IMS exit lens voltage is fixed for maximum transmission, recording the full ion mobility spectrum with three example structures, (b) during ion mobility slicing, the IMS exit lens voltage is kept low to prevent ion transmission and raised after a time *d* (relative to the IMS start trigger) for a time *w*. This transmits only ions during the time the voltage is high. In the illustrative mobility spectrum, the blue conformation is transmitted while the green and red species are removed.

Ion mobility slicing is performed using the IMS Exit lens (indicated by B in Figure 1). In normal operation (both MS^*n*^ and IM-MS) the lens is kept at a fixed voltage corresponding to the maximum ion transmission. To perform mobility slicing, the lens is held at a lower potential to prevent ion transmission. The voltage is raised to allow transmission of ions of a given mobility; see Figure 2. The IMS start signal is used as a start trigger for a programmable delay (*d* in Figure 2) after which the lens is set high for a selected duration (*w* in Figure 2), allowing control over the mobility window to be transmitted (via control of the delay of the sliced ions).

### Vacuum Chamber for Spectroscopy

Low pressures and long trapping times are essential to perform IR photofragmentation spectroscopy. Therefore, the design of our Photo-Synapt differs from the Synapt G2 modified by the group of Perdita Barran,^22,23^ where irradiation experiments were performed within the trap or transfer TWave. In the Photo-Synapt, the spectrometer was extended with the addition of a vacuum chamber between the transfer TWave and TOF entrance optics (C in Figure 1 and S2 for the complete pumping system).

This new chamber provides both the required space to perform ion trapping and irradiation and the differential pumping stage required to achieve a controllable pressure low enough to perform efficient IR multiphoton dissociation (IRMPD) (down to 10^–6^ to 10^–7^ mbar).

### Ion Optics Overview

The top-inset in Figure 1 shows a zoomed-in illustration of the additional ion optics in the Photo-Synapt for trapping, irradiation, and manipulation of ions. The two hexapoles (Hex_1_ and Hex_2_) function as ion guides in transmission mode (i.e., when there is no trapping), with no drop in performance in either MS or IM-MS modes.^20^

In trapping mode, the hexapoles function as ion traps in combination with pulsed lenses *L*_1_ (transfer TWave exit lens), *L*_2_ (new lens), and *L*_3_ (first acceleration electrode). Increasing the voltage on *L*_2_ and *L*_3_ by a few *V* relative to the hexapole rods will trap ions within the hexapoles. *L*_1_ is typically operated at a voltage that allows ions from the source to enter Hex_1_, but its voltage is high enough to prevent ions exiting Hex_1_ after collisional cooling. This allows us to continuously fill Hex_1_ during measurements, resulting in fewer ion losses.

Each hexapole contains a pin trap (see inset at the bottom of Figure 1), which is composed of 3 sets of pins inserted between the hexapole rods of both Hex_1_ and Hex_2_. The first set of pins (*P*_1_ and *P*_2_) are used to transfer ions from the pin traps to Hex_2_ or the TOF, respectively.

The second and third sets of pins (*T*_1_ and *T*_2_) are biased with a small negative voltage relative to the hexapole DC offset. Ions will undergo collisional cooling within the hexapoles, leading them to become trapped in *T*_1_ and *T*_2_. *T*_1_ is composed of 2 sets of 6 pins, while *T*_2_ is composed of 2 sets of 4 pins. For *T*_2_ a pair of pins have been removed to enable optimal overlap of the ion cloud and laser beam.

### Acquisition Cycle

**Figure 3:**
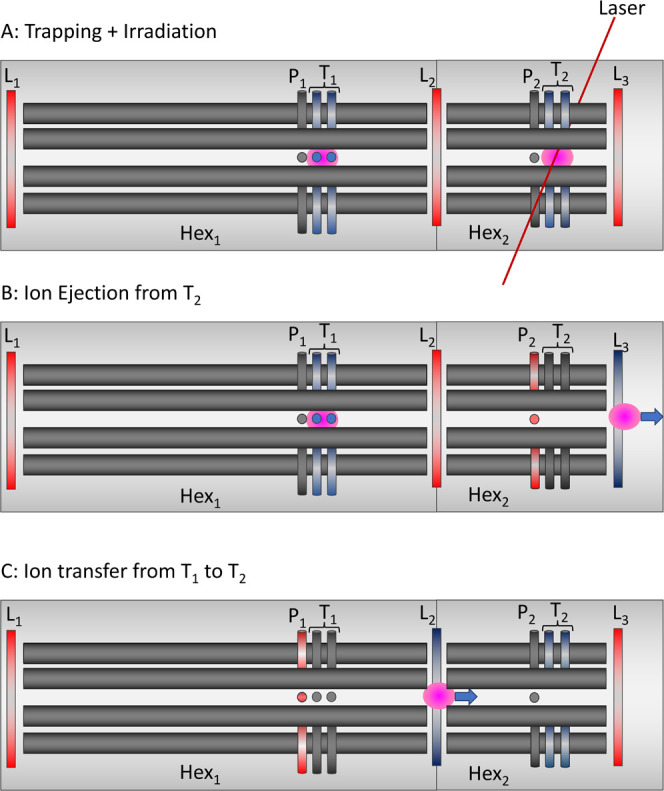
Schematic of the different voltage settings used on trapping electrodes during a single cycle showing trapping and irradiation (A), ejection of ions from trap 2 (B) and transfer of ions between trap 1 and trap 2 (C). Typical voltages can be found in Figure S5. A red color indicates a positive voltage (relative to the DC offset of the hexapole), a blue voltage a negative voltage. The pink circle represents the ion cloud.

The Photo-Synapt is operated in three different states during an acquisition cycle, which we term trapping (and irradiation), ejection, and transfer (see Figure 3 and Section S1 of the Supporting Information). Ions undergo a complex sequence of manipulations during this cycle, which can be summarized by the following steps: (i) accumulation of ions in Hex_1_ followed by collisional cooling into *T*_1_, (ii) transfer from *T*_1_ into Hex_2_ and subsequent collisional cooling into *T*_2_, (iii) irradiation in *T*_2_, and (iv) ejection to the pusher and detection. To maximize the duty cycle, accumulation of ions in the first trap (*T*_1_) and irradiation in the second trap (*T*_2_) are performed simultaneously. This results in a shorter acquisition time and fewer wasted ions. These individual steps are discussed in further detail in the Supporting Information (SI.1), and here, we summarize what happens to the ions during each instrument state.

### Trapping

In the trapping mode, *L*_2_ is closed, and the two hexapoles act as independent traps. *L*_1_ is kept at a voltage that allows ions to enter Hex_1_ but prevents them from exiting, leading to constant filling of Hex_1._ Hex_1_ is operated at a pressure of between 10^–3^ and 10^–5^ mbar, that is, ions undergo collisional cooling into *T*_1_. At the same time, ions that have been transferred to Hex_2_ undergo collisional cooling into *T*_2_ and are irradiated with either IR or UV laser pulses.

Ions can be stably trapped in this state for up to 10 s, which is only limited by internal MassLynx settings. When using ion mobility slicing, the same conditions are used, with the difference being that the filling of Hex_1_ is now linked to the number of ion mobility cycles that occur during the time of the trapping, as ions only reach Hex_1_ when the mobility slicing lens is open.

### Ejection

During the ejection state, ions are ejected from *T*_2_ toward the TOF to be analyzed. This is achieved by simultaneously removing the trapping potential on *T*_2_, adding a positive potential to *P*_2_, and setting *L*_3_ to an open state, see Figure 3b. The TOF pusher is triggered after a set delay, depending on the *m*/*z* of the ions, and subsequently, a mass spectrum is recorded. As photofragmentation results in ions with a range of *m*/*z* values being present in *T*_2_, ions will arrive at the pusher at different times (see the Supporting Information, Figure S3). Thus, to record all ions, several different pusher delays must be used within a single acquisition cycle.

### Transfer

During the transfer state, ions are transferred from *T*_1_ toward Hex_2_. To achieve this, a positive pulse is applied to *P*_1_ and *L*_2_ is lowered to allow ions to pass, see Figure 3c. During this time, *L*_1_ remains open to continue to fill Hex_1_, but ions are blocked from entering Hex_2_ by *P*_1_. As with the situation mentioned above, the time it takes ions to reach Hex_2_ from *T*_1_ will depend on the *m*/*z*, see Figure S4. However, in this case, *T*_1_ typically contains ions of a single *m*/*z*, and thus a single transfer time can be used throughout the acquisition cycle. Once the transfer of ions has been achieved, the electrodes return to the trapping state and this cycle repeats.

## Methods

### Sample Preparation VEALYL

Aliquots of the hexapeptide VEALYL, a peptide segment from the B chain of insulin (purity >95%, GeneCust) were dissolved in 50:50 v/v water/methanol at a concentration of 200 μM.^9^ These samples were prepared on the day of measurement, loaded in gold-coated nanoelectrospray emitters and introduced into the Photo-Synapt using nano-ESI in positive ion mode. Source and IM-MS parameters that were used: capillary voltage at 0.35 kV, sampling cone at 45 V for the 2^1+^ and 15 V for the 4^2+^ and the 6^3+^, desolvation temperature of 150 °C, source temperature of 80 °C, trap gas flow 2 mL/min, helium gas flow 180 mL/min, IMS gas flow 40 mL/min, IMS wave velocity 400 m/s and the IMS wave height at 20 V.

### Sample Preparation Glycan Fragments

Human alpha-1-acid glycoprotein (hAGP) stock solution (1 mg/mL in 100 mM ammonium acetate buffer) was diluted to 50 μg/mL in water/acetonitrile/formic acid (1:1:0.05 v/v/%). Transferrin (TF) was diluted to 100 μg/mL in water/acetonitrile/formic acid (1:1:0.05 v/v/%). Both hAGP and TF were directly infused using electrospray ionization (120 μL/h) in positive ion mode. For ion mobility separation an IMS gas flow of 90 mL/min was used, a wave velocity of 650 m/s and a wave height of 40 V.^39^

#### Laser

A tunable IR laser (FireFly IR, M-Squared) was used for the reported IR spectroscopic experiments. The laser is tunable between 2700 and 4000 cm^–1^ has an output power of more than 80 mW, pulse durations of <10 ns, and was operated at a 150 kHz repetition rate.

#### Data Processing

MassLynx V4.1 (MassLynx software instrument control) was used to obtain the mass and mobility data; Origin 2023 was used to analyze and plot the data.

## Results and Discussion

### IR Action Spectroscopy of Mass- and Mobility-Selected VEALYL Oligomers

The peptide VEALYL, a segment from the B chain of insulin, was selected to illustrate the capabilities of the Photo-Synapt to create IR spectra of mass- and mobility selected oligomers. This six-residue sequence has a high propensity to form amyloid aggregates and therefore has been the topic of many studies.^14,40,41^

**Figure 4:**
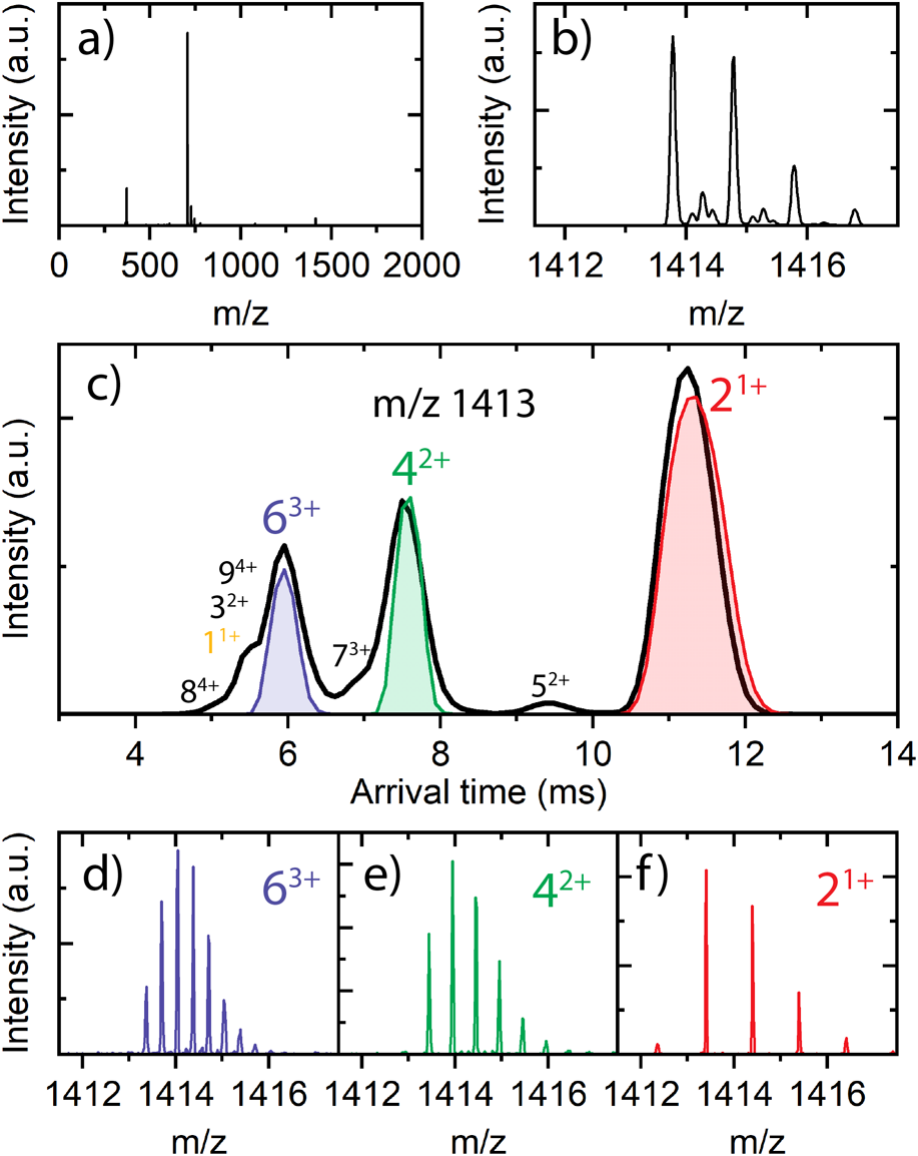
(a) Full mass spectrum of freshly prepared VEALYL; (b) zoomed-in mass spectrum of quadrupole mass filtered *m*/*z* 1413; (c) arrival time distribution of quadrupole selected *m*/*z* 1413 of VEALYL, the experimental arrival times of the oligomers are annotated and in color; (d–f) the obtained mass spectrum after slicing and trapping of the 6^3+^, 4^2+^, and 2^1+^, respectively.

Figure 4a shows the mass spectrum of freshly prepared VEALYL (full MS data can be found in Figure S6). The dominant peak at *m*/*z* 707 in the mass spectrum originates from the singly charged monomer (1^1+^), or more precisely from [*n*]^*n*+^ type oligomers with *n* being the number of monomeric units. Furthermore, the doubly charged monomer adducted with potassium ([1 + H + K]^2+^) is present at *m*/*z* 373 and a small peak is observed around *m*/*z* 1413, which could correspond to [2*n*]^*n*+^ oligomers, such as the 2^1+^, 4^2+^, and 6^3+^ species. Figure 4b displays a zoomed-in portion of the *m*/*z* 1413 quadrupole filtered mass spectrum, indicating the presence of more isobaric species present than just the 2^1+^ dimer. Subsequently, we have employed ion mobility on the quadrupole selected oligomers, which allows the separation and identification of individual oligomers with the same *m*/*z* ratio; see Figure 4c. To assign the mobility peaks, the isotopic distribution of the extracted mass spectra of each mobility peak is evaluated; see Figure 4d–f.

The most intense peak in Figure 4c (red) originates from the mobility of the dimer singly charged (2^1+^), the consecutive two other intense peaks (highlighted in green and blue respectively) result from the doubly charged tetramer 4^2+^ and the triply charged hexamer 6^3+^, followed by a small peak originating from the 8^4+^ oligomer. Even though the quadrupole mass filter was used to select only [2*n*]^*nz*+^ oligomers with *m*/*z* 1413, several other signatures appeared in the mobility spectrum, such as the 1^1+^ and in lower abundances 3^2+^, 5^2+^, 7^3+^, and 9^4+^ oligomers. These oligomer fragments result from fragmentation of the quadrupole selected [2*n*]^*n*+^ oligomers after the mass filter and before the ion mobility separation (i.e., in the trap cell). Here, our main goal was to get high transmission of the oligomers; therefore, all parameters were set for optimal transmission, at the cost of some fragmentation in the trap cell. By using the ion mobility slicing, these unwanted fragments are removed.

With quadrupole and ion mobility selection combined, only the oligomer ions with specific *m*/*z* and selected mobility are transferred into the traps (*T*_1_ and *T*_2_), as indicated by the colored traces in Figure 4c. Consequently, the mass spectra presented in Figure 4d–f result only from species that are stored in the traps, as other ions are selectively filtered out by either the quadrupole mass filter or mobility slicing. Subsequently, the selected and trapped oligomer ions are irradiated for about 400–1000 ms in trap *T*_2_. The resulting photofragments are transferred and analyzed at the TOF. By repeating this experiment over the entire IR wavelength of interest, we can record their IR action spectrum. To plot such an IR action spectrum, the photofragmentation per wavelength is calculated using
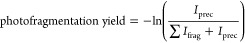
1with ∑*I*_frag_ the sum of the intensity of all the fragments and *I*_prec_ the intensity of the precursor ion. This method of calculating the photofragmentation yield makes it possible to linearly correct for the irradiation time and the laser power.^42,43^ IRMPD results in general in similar fragmentation pathways as collision induced dissociation (CID), where the weakest bond is broken; see the Supporting Information, Figure S7a,b. Determining the IR photofragmentation yield, and thus plotting the IR spectrum, can be quite challenging when studying an aggregation process. Formed oligomer-photofragments of larger oligomers that are stable in the trap and can absorb the IR radiation as well. In the case of a multiple charged oligomer-photofragment, for example, 4^2+^ from the 6^3+^, secondary fragmentation of the 4^2+^ can lead to the formation of two singly charged monomers. This could result in overestimated yield at wavelengths where the photofragment itself absorbs more than the precursor. Therefore, it is important to carefully compare the spectra obtained by including all fragments with the spectra involving only the primary fragments, to determine whether the yield has altered.

For the 2^1+^ oligomer, both CID and IRMPD at 2970 cm^–1^ show that this oligomer fragments exclusively into the 1^1+^*m*/*z* channel. Therefore, we can use the intact singly charged monomer (1^1+^) as the fragment to calculate the photofragmentation yield (1). The 4^2+^ species could theoretically fragment into a trimer, dimer, and/or monomer; however, CID (Figure S7c) shows that the 4^2+^ oligomer exclusively fragments into the 2^1+^ species, which is confirmed by the IRMPD data presented in Figure S7d. Of course, the photofragmentation routes can become more complex for the higher-order oligomers, which is clearly illustrated by the 6^3+^ oligomer (see Figure S7e,f). The CID spectrum shows the simultaneous fragmentation of the 6^3+^ oligomer into 5^2+^, 4^2+^, and 2^1+^*m*/*z* channels, while the IR data suggests that the 4^2+^ photofragment is formed before the 2^1+^ photofragment. To determine the photofragmentation yield, we have included all the above-mentioned fragments, including the 1^1+^. The individual intensities of the precursor ion, which have overlapping *m*/*z* values for our oligomer series (2^1+^, 4^2+^ and 6^3+^), are derived from their expected isotopic ratios (see the Supporting Information Section S2, Figures S8, S9, and S10 and Table S1 for details). Although an approximation, it reflects the reality more accurately than when the full *m*/*z* = 1413 range was taken as precursor (as the formed photofragments can also appear in this *m*/*z* channel).

**Figure 5:**
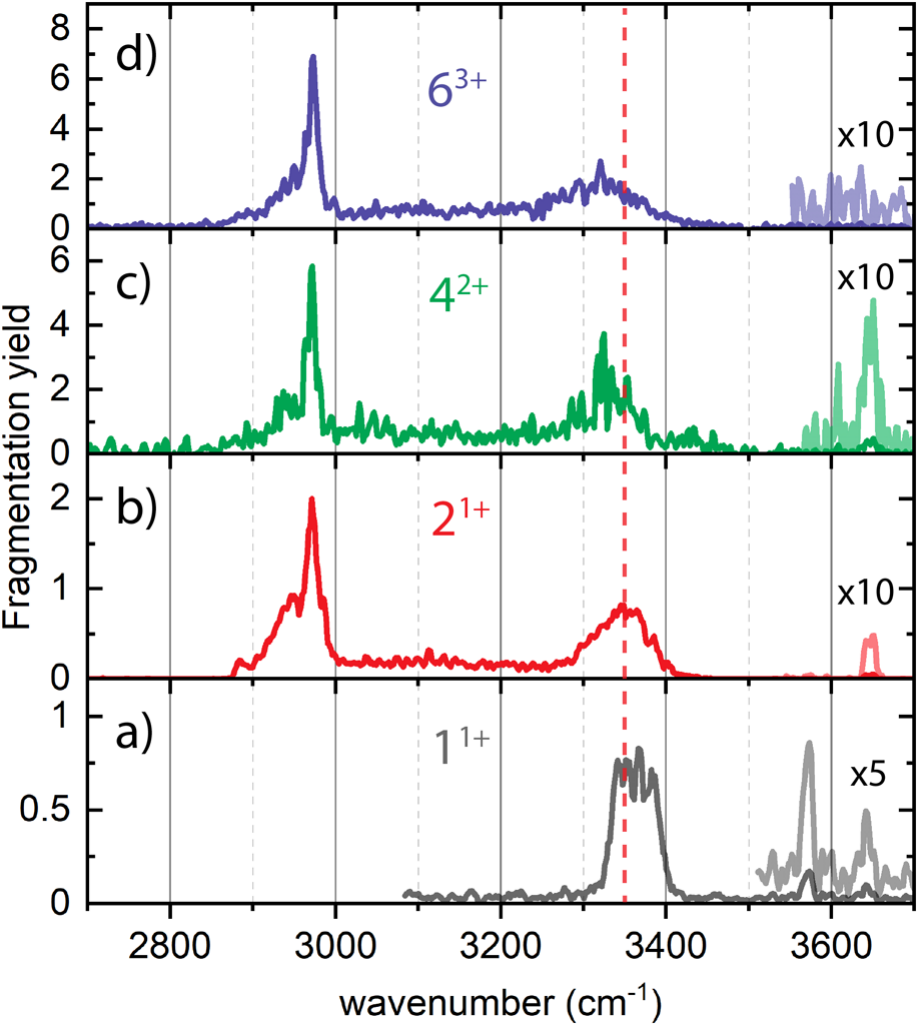
IR photofragmentation spectra of the (a) 1^1+^ (gray), (b) 2^1+^ (red), (c) 4^2+^ (green) and (d) 6^3+^ (blue) between 2700 and 3700 cm^–1^. The red dashed line shows the peak position of the 2^1+^ and highlights the redshift in the spectra of the 4^2+^ and 6^3+^.

The mass- and mobility-selective IR photofragmentation spectra of the 2^1+^, 4^2+^, and 6^3+^ oligomers are measured in the X–H stretching region (with X = C, N, or O) from 2700 to 3700 cm^–1^ (Figure 5b–d) and plotted together with the singly charged monomer (1^1+^) for comparison (Figure 5a). The spectra are all averaged over >5 individual scans and subsequently smoothed (raw data is presented in Figure S11 together with the singly charged monomer). Scans with different irradiation times were linearly corrected to match each other. The spectra of 2^1+^, 4^2+^, and 6^3+^ are built up in a similar way: a weak OH stretch vibration at 3646 cm^–1^ (not observed for 6^3+^), indicating a free OH stretch vibration, originating from the Tyr-OH;^44^ a broad feature between 3250 and 3400 cm^–1^, which can be attributed to hydrogen bonded N–H stretching vibrations; a strong and sharp feature at 2970 cm^–1^, and a smaller and broader peak at 2940 cm^–1^, both likely C–H stretch vibrations. Additionally, there is activity between 3000 and 3300 cm^–1^ in the form of a broad and featureless elevated yield.

Although the IR spectra look at first glance quite similar, they differ clearly in the N–H stretch region. In Figure 5b, the 2^1+^ shows a broad peak between 3300 and 3400 cm^–1^ with its maximum around 3350 cm^–1^ (indicated with the red dashed line), red-shifted by 15 cm^–1^ with respect to the 1^1+^ (Figure 5a). With increasing aggregation, this NH band continues to broaden and shift to lower wavenumbers (Figure 5c,d), with 6^3+^ showing strong features down to 3250 cm^–1^. The 1^1+^ spectrum shows an additional peak at 3573 cm^–1^, which is not present in the other spectra. This indicates that the c-terminal –OH and/or the glutamic acid –OH are likely involved in hydrogen bonding upon aggregation. The combination of both mass and mobility selections makes it possible to obtain IR spectra of isobaric oligomers, which allows for more detailed experimental research into oligomerization and fragmentation pathways of peptides.

### IR Signatures of Isomers of Glycan Fragments

Proteins can exhibit intricate glycan structures that shape their physical properties, half-life and receptor recognition.^45^ Understanding these interactions requires knowledge of the exact glycan structure as different isomeric features can lead to different biological activities. Sialic acid linkages play a crucial role, as many glycan structures are terminated with sialic acid, making it an easily accessible glycan epitope for biomolecular interactions. The most common sialic acid linkages are the α2,3 and α2,6 isomers which are relevant in human virus entry or cancer biology.^46−48^ Despite their biological significance, the analysis of these isomers presents an analytical challenge. Recent studies have shown that IR-MS enables direct identification of sialic acid linkage.^31,49−53^ The fragmentation of sialylated glycans yield characteristic oxonium ions, such as the sialyllactose trisaccharide at *m*/*z* 657 which exhibits distinct ion mobility features depending on sialic acid linkage.^54,55^

**Figure 6:**
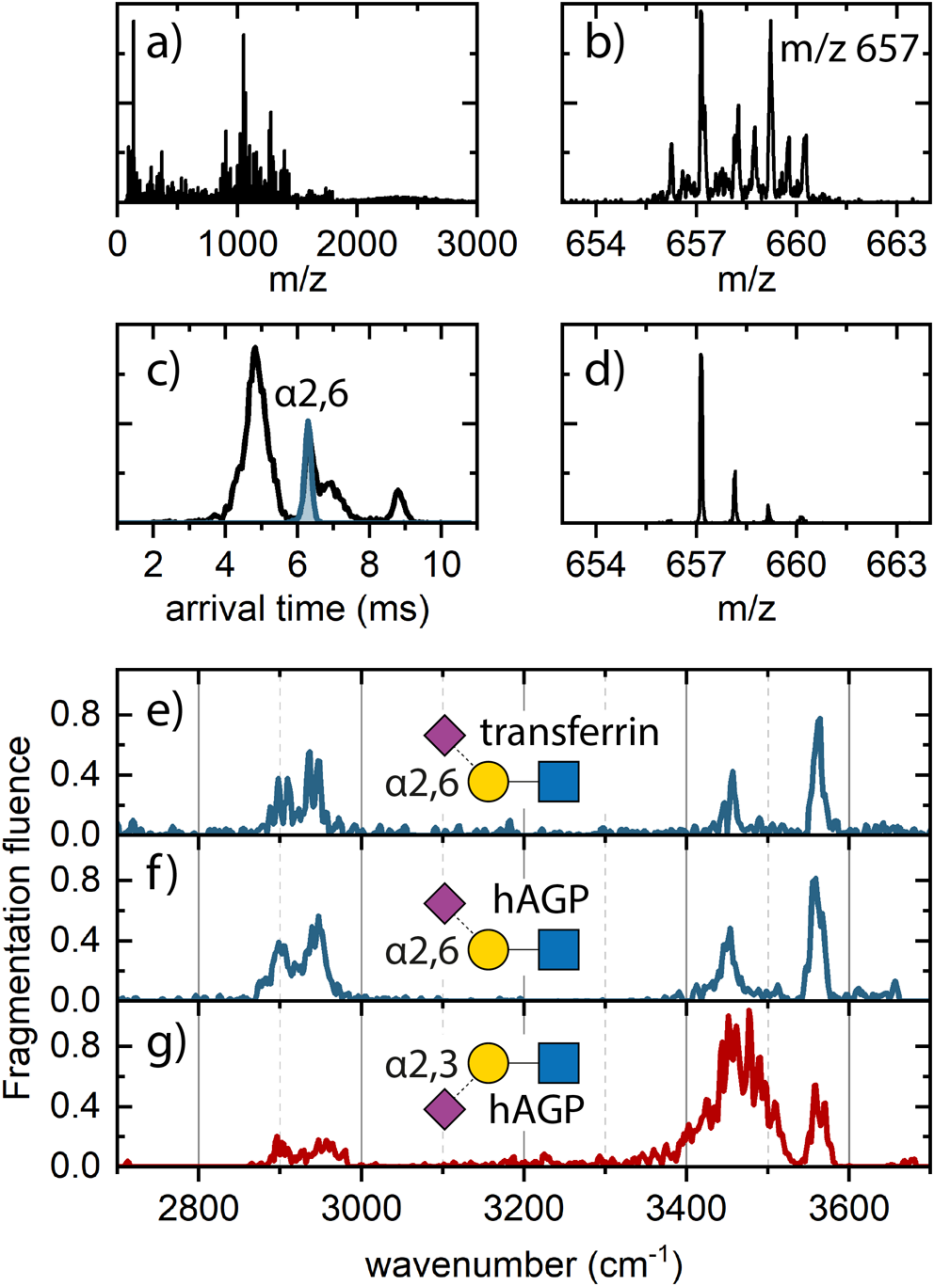
(a) Full mass spectrum of TF under harsh source conditions, blue arrow indicates the location of the *m*/*z* 657 fragment, (b) quadrupole filtered mass spectrum of *m*/*z* 657, (c) full arrival time distribution (black) for the mass filtered *m*/*z* 657, blue indicates the experimental sliced section corresponding to the α2,6 isomer of TF, (d) mass spectrum of the mass filtered, and ion mobility sliced *m*/*z* 657 fragment. Quadrupole selected (*m*/*z* 657), and IM sliced IR spectra of (e) TF α2,6-linked sialic acid isomer, (f) human-alpha-glycoprotein α2,6, and (g) human-alpha-glycoprotein α2,3 isomer.

We use this sialylated *N*-glycan fragment (*m*/*z* 657) to demonstrate the possibility of the Photo-Synapt to obtain mass and ion mobility selected IR spectra from mixtures of α2,6- and α2,3-linked sialic acid isomers that are directly extracted from glycoproteins. Therefore, the full glycoproteins, that is, TF and hAGP, were dissolved and introduced in the mass spectrometer using nano-ESI in positive mode. Employing harsh source conditions (2.1–2.3 kV capillary voltage, 90–130 V sampling cone), the glycoprotein significantly fragmented, resulting in the *m*/*z* 657 B3 fragments among other, see Figure 6a.

Since TF predominantly holds the α2,6 sialylated fragment (α2,6:α2,3 = 95:5), TF will provide a clear IM and IR signature for the α2,6 isomer. Figure 6a–d shows the workflow for generating, mass selecting, and mobility slicing the α2,6 isomer of TF. Using the quadrupole mass filter, the sialylated *m*/*z* 657 fragment was isolated, indicated by the arrow in the full mass spectrum in Figure 6a, resulting in the mass spectrum presented in Figure 6b. The signature is a combination of residual fragments of the TF protein combined with a sialylated *N*-glycan fragment. The ion mobility spectrum was recorded of the quadrupole selected *m*/*z* 657 region was recorded (see Figure S12a), and the mobility peak corresponding to the α2,6 isomer was identified by analyzing the mobility extracted mass spectra, see the Supporting Information, Figure S12c–f. Subsequently, ion mobility slicing was used to isolate the α2,6 isomer (Figure 6c, highlighted in blue), resulting in a clean mass and ion mobility selected mass spectrum of the *m*/*z* 657 fragment. Finally, the mass and mobility selected IR spectrum of the B3 fragment originating from the TF glycoprotein was recorded; see Figure 6e.

The same process was used for human α1-acid-glycoprotein (hAGP), which is shown in detail in Figure S13 of the Supporting Information. In contrast to TF, both α2,6:α2,3-isomers are present in hAGP, with the ratio depending on the antenna type.^39,54^ Here, for the direct fragments from the full hAGP protein, we observe a 78:22 ratio for α2,6: α2,3 isomeric ratio, indicated by the labels V and VI in Figure S13c. We recorded the mass and mobility selected IR spectra of the B3 fragments originating from both the α2,6 isomer (Figure 6f, blue trace) and the α2,3 isomer (Figure 6g, red trace) from hAGP.

To record the IR spectra of the *m*/*z* 657 B3 fragment ions, we trapped and irradiated the ions for 2 s, after which we detected the precursor mass and the fragment mass (*m*/*z* 366) using two different time delays. The photofragmentation yield is calculated via eq 1 and the resulting spectra are plotted in Figure 6e–g. The raw data are presented in Figure S14. The α2,6 isomer of hAGP shows an IR spectrum identical to that of TF (Figure 6e) which confirms that both species are the α2,6 isomer. The α2,3 isomer, with a slightly later ion mobility arrival time, clearly shows a dissimilar IR spectrum compared to the α2,6 isomer. Even though the peak position at 3560 cm^–1^ is similar for both isomers, the region between 3400 and 3530 cm^–1^ is visibly different (all attributed to OH stretch vibrations). Where the α2,6 isomer shows a single intense peak at 3450 cm^–1^ and some minor activity around 3500 cm^–1^, the spectrum of the α2,3 isomer shows a broad band covering a wide region. Additionally, the ratio in peak intensity between 3560 and 3450 cm^–1^ is reversed, with the α2,3 isomer showing more intensity in the lower wavelength region. The CH pattern observed below 3000 cm^–1^ is quite similar for both isomers with wo main features present at 2900 and 2942 cm^–1^. Although, we have measured the sialyllactose trisaccharide cleaved from full glycoproteins compared to the isolated 3′- and 6′-sialyl-*N*-acetyllactosamine by Depraz Depland et al.,^51^ a similar trend is observed in the IR spectra; the α2,3 isomer shows a very broad spectrum in the OH range, while the α2,6 isomer results in resolved spectra.

The combination of both *m*/*z* selection and ion mobility slicing results in recording unique IR spectra and allows us to record and identify sialylated *N*-glycan fragments from glycoproteins without any preseparation technique via their unique IR spectra.

## Conclusions

In this paper, we have demonstrated the strength of combining ion mobility, mass spectrometry, and IR action spectroscopy in one single experiment. Therefore, we have developed and applied a three-dimensional hyphenated mass spectrometer: the Photo-Synapt. This combination makes it possible to select a specific analyte with mass spectrometry and subsequently to separate a conformational structure with ion mobility and record the specific vibrational signatures of the mass and mobility selected species by IR action spectroscopy.

The Photo-Synapt, is initially developed to probe higher order transient oligomers; important species along the aggregation pathway of peptides and proteins related to neurodegenerative diseases. Employing the Photo-Synapt, we followed the oligomer formation in the [*n*]^2*nz*+^*m*/*z* channel. By selecting this oligomer type with a quadrupole mass filter, then separating and identifying the present oligomers ranging from singly charged dimers to quadruply charged octamers within this *m*/*z* channel using ion mobility and employing mobility slicing to isolate a specific isomer, we have been able to observe unique IR features in the NH stretching vibrational region for each oligomer. Furthermore, combining *m*/*z* selection with ion mobility slicing enables the direct identification of the linkage of sialylated *N*-glycan fragments from glycoproteins through their distinctive OH signatures in their IR spectra, eliminating the need for preseparation techniques. Together, these two examples illustrate the wide versatility of the Photo-Synapt.

## Data Availability

The data underlying this study are openly available in DataCite Commons at https://doi.org/10.48338/VU01-GGJP9S.

## Supporting Information Available

Supporting Information additional experimental details, raw data and detailed clarifying explanations on the instrumentation and data acquisition (PDF)
