# Supplementary material for: Probing High-Order Transient Oligomers Using Ion Mobility Mass Spectrometry Coupled with Infrared Action Spectroscopy
Source: Anal Chem. 2024 Aug 16;96(34):13962–70. doi: 10.1021/acs.analchem.4c02749 (PMC11359389; doi:10.1021/acs.analchem.4c02749)
Supplement: Supplementary file 1 — ac4c02749_si_001.pdf [file ac4c02749_si_001.pdf]

Supporting Information of:

Probing High-Order Transient Oligomers using Ion Mobility Mass Spectrometry coupled to Infrared Action Spectroscopy

Sjors Bakels[1,2], Steven Daly[3], Berk Doğan[3], Melissa Baerenfaenger[1,2], Jan Commandeur[3], and Anouk M. Rijs[1,2]*

1 Division of Bioanalytical Chemistry, Department of Chemistry and Pharmaceutical Sciences, Amsterdam Institute of Molecular and Life Sciences, Vrije Universiteit Amsterdam, De Boelelaan 1105, 1081 HV Amsterdam, The Netherlands.

2 Centre for Analytical Sciences Amsterdam, 1098 XH, Amsterdam, The Netherlands.

3 MS Vision, Spectrometry Vision B.V., Televisieweg 40, Almere 1322 AM, The Netherlands

Table of contents

Contents

Figure S1. Pressure sleeve. 2

Figure S2. Pumping system. 3

Section S1. Trapping modes in detail. 4

Figure S3. Normalized intensity versus pusher delay. 7

Figure S4. Normalized ion count versus transfer time 8

Figure S5. Schematic representation of voltages on the hexapole pin traps. 9

Figure S6. Full MS of VEALYL. 10

Figure S7. Fragmentation pathways for VEALYL using CID and IRMPD. 11

Section S2. Solving overlapping m/z species of VEALYL 12

Figure S8. Theoretical and experimental isotopic distributions of VEALYL 4^{2+}. 13

Figure S9. Theoretical and experimental isotopic distributions of VEALYL 6^{3+}. 13

Table S1. Theoretical isotopic distributions of VEALYL 4^{2+} and 6^{3+}. 14

Figure S10. Mass resolution after transmission and trapping 15

Figure S11. Raw IRMPD data for VEALYL oligomers. 16

Figure S12. Ion mobility analysis for glycan fragment m/z 657 of Transferrin (TF). 17

Figure S13. Ion mobility analysis for glycan fragment m/z 657 of hAGP. 18

Figure S14. Raw IR data for m/z 657. 19

Figure S1. Pressure sleeve.

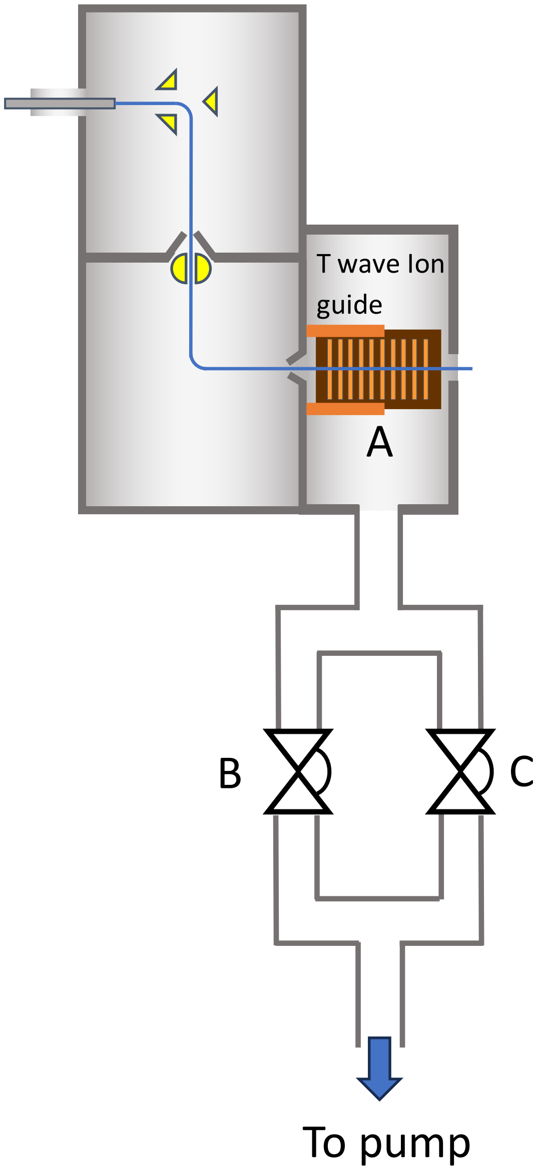

Figure S1. Modification of the transfer region of the Photo-Synapt to improve transmission of ions from atmospheric to pressures compatible with ion optics. Both pressure sleeve and throttle valves restrict pumping, yielding high local pressures and more collisional cooling. This allows high m/z ions to be 'grabbed' by the rf voltages on the ion guide. A Peek pressure sleeve (A) is fitted over the T-wave ion guide to restrict the pump out of gas entering through the extraction cone and create higher pressure. In addition, the gas line to the backing pump is modified by addition of two throttle valves (B (NW10) and C (NW25)). In normal operation, valve B is kept open to allow full pumping capacity. The pressure in the desolvation region can be further increased by closing B, and partially closing C.

Figure S2. Pumping system.

We have moved the nEXT 240 pump from the transport region to the new chamber, and replaced the transport region pump by an EXT 255 pump. The new pump is integrated into the existing roughing line, supplied with its own 24V power supply, and integrated into the pump control interface of the Photo-Synapt, so it can be started/stopped with the other pumps via MassLynx.

The pressure in both the new chamber and the transport regions, containing trap 1 and trap 2 respectively, have their pressure controlled via a gas inlet. On the air side this inlet is connected to a short piece of peek which acts as a restriction to limit the flow of gas. The restriction is connected to a N2 line, which is typically held at 1 bar. The pressure is typically switched from a resting state (1e-5 mbar for T1, 1e-7 mbar for T2) to a trapping state (1e-4 mbar T1, 1e-6 mbar T2) via a manual valve. Fine pressure control can be attained by changing N2 pressure via the regulator.

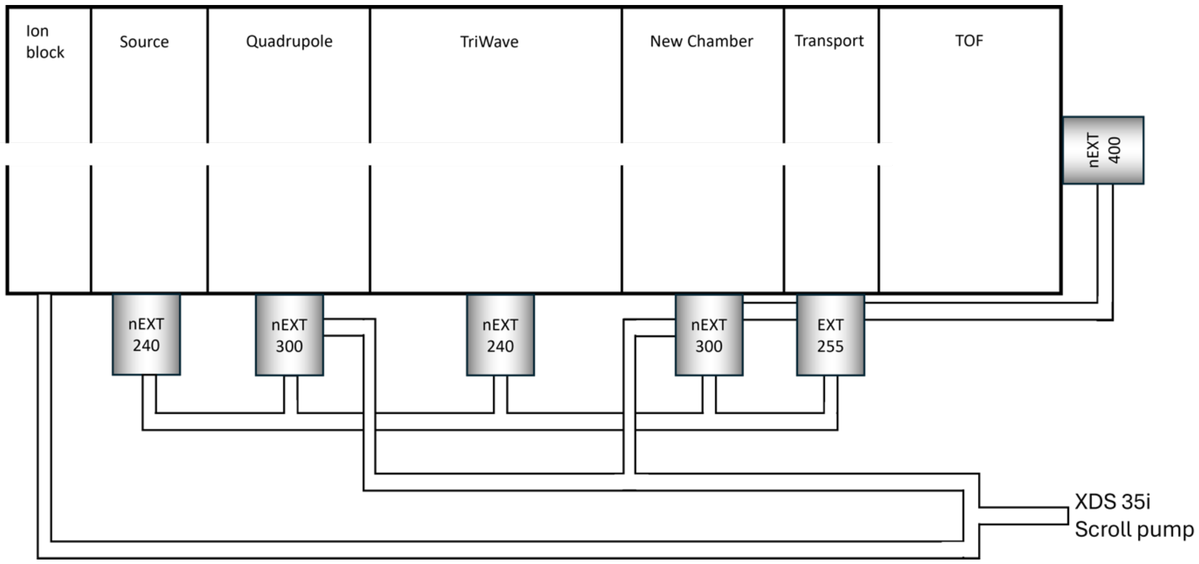

Figure S2. Pumping system of the Photo-Synapt.

Section S1. Trapping modes in detail.

Ions undergo a complex cycle of trapping which can be summarized by the following steps: (i) accumulation in Hex_1 followed by collisional cooling into T_1, (ii) transfer from T_1 into Hex_2 and subsequent collisional cooling into T_2, (iii) irradiation at T_2, and (iv) ejection to the pusher and detection. To maximize the duty cycle, accumulation of ions in the first trap and irradiation in the second trap are performed simultaneously, resulting in shorter acquisition time and fewer wasted ions. In the main text, we presented the different states of the mass spectrometer and what each is used for. Here we will follow a bunch of ions on their journey through the mass spectrometer to give a better understanding of what is happening to the ions.

(i) Accumulation in Hex_1

Typical voltages on the lenses during trapping are shown Figure S5 (blue) referenced to the DC offset of Hex_1. The voltage on L_1 is kept low enough that ions can continuously enter the hexapole, but high enough that they are unable to exit once they have undergone collisional cooling within the hexapole. This voltage is typically 0.5V above the static offset, which is -180V in the Photo-Synapt. The DC offset of the hexapoles is typically kept within 1 V of the static offset. L_2 is kept at a voltage that ensures 100% reflection of all ions. This can be confirmed by setting all other voltages to zero and increasing the voltage on L_2 until no ion transmission is observed.

The negative voltage applied to T_1 produces a second potential well within the hexapole. Ions are constantly undergoing collisional cooling in the hexapole (ambient pressure is between $10^{-3} – 10^{-4}$ mbar N_2). If a collision occurs while the ions are traversing T_1, then it is possible to remove enough energy that they are trapped within this potential well. This occurs on the 10s of millisecond timescale. The exact voltage on T_1 is somewhat mass dependent and can be quite sensitive to changes of 0.1 V, and thus is tuned for each species. Ions can be trapped for up to 10 seconds, where the limit is imposed by software.

When performing ion mobility slicing in combination with trapping, the process is essentially the same. The only difference is in the determination of the trapping time (accumulation time). Without ion mobility slicing, the trapping time can be determined arbitrarily as ions are always entering the hexapole from the source. In ion mobility slicing mode, ions will arrive in discrete packages. The Photo-Synapt records ion mobility by binning 200 ToF acceleration pushes following the IMS Start signal. Hence the ion mobility cycle time is mass dependent as the time between pushes also increases with mass. So, the trapping time when using ion mobility slicing must be some integer number of the ion mobility cycle time and will be defined as X number of cycles. This leads to the same trapping dynamics but is simply an additional factor to consider when studying mobility sliced ions.

(ii) Transfer from Hex_1 to Hex_2:

Ions that have been trapped in Hex_1 must be transferred to Hex_2 where they will be irradiated. Typical voltages used during transfer are shown in Figure S5 (red). After the desired trapping time, the trapping voltage on T_1 is removed, and a positive voltage is applied to P_1. The voltage on P_1 serves two purposes: it must provide a push to the ions to give them kinetic energy to move towards Hex_2, and must be high enough to prevent ions from the source passing directly into Hex_2 (as L_2 must be open during the transfer step). The voltage required to block ions can be checked by setting all other voltages to 0V and varying P_1 until no ions are transmitted. At the same time, L_2 is lowered to allow ions to pass. It is typically found that a slightly negative potential is better for ion transmission, as this helps ions negotiate the fringe fields around the hexapoles. Once ions are within the second hexapole L_2 is closed again, and the voltages are returned to the trapping state.

As we are pushing ions, the transfer time t depends on the voltage and m/z as $t \propto \sqrt{\left(\frac{m}{z}\right)}/E$. Assuming a fixed voltage on P_1 (which is how the instrument is operated, the value is set to block ions rather than optimized for time), the time which L_2 must be open depends on mass-to-charge of the ions. In this case we will almost always be dealing with ions of a single m/z and so we can optimize transfer time based on ion signal, an example is shown in Figure S4. At short times below 50 μs no ions are seen as L_2 is raised before ions have time to enter the second hexapole. After these 50 μs, the ion cloud has time to pass into Hex_2 as evidenced by the increase in intensity. In this case, an optimum transfer time of 130 μs is found, which is when the ion cloud is primarily inside Hex_2. At longer times, the ions cross back into Hex_1, leading to a loss of signal. The transfer time is optimized in this manner for each different species to ensure we have best transmission between hexapoles.

(iii) Trapping and irradiation in Hex_2.

Ions are trapped within the hexapole for which the typical voltage are highlighted in blue in Figure S5 and undergo collisional cooling into the second pin trap. Due to the small size of Hex_2, it is not possible to measure the trapping time experimentally as in Figure S4. However, ion optic simulations suggest that trapping times on the order of tens of milliseconds are required for ions to enter T_2. The longer trapping time is due to the lower operating pressure of Hex_2, which is typically between $10^{-5} – 10^{-7}$ mbar. However, since ions arrive in Hex_2 as a single ion bunch, this means the majority of ions are within T_2 after 100 ms, which is much shorter than our typical irradiation times. T_2 behaves similarly to that of T_1, and there are no observed differences in the trapping with the removal of two pairs of pins to allow laser access.

The voltage of L_3 during trapping and irradiation must be set very carefully. Since the lens is close to the ion trap, if the voltage is set too high then it can influence the position of the ions within T_2, pushing the ion cloud away from the center of the trap. Thus, L_3 is typically set to the lowest value which allows for trapping to minimize its influence on the ion cloud during trapping.

As noted, T_2 has two pairs of electrodes removed to allow to perpendicularly couple a laser. Perpendicular coupling is performed via a pair of CaF_2 windows centered on T_2. Perpendicular irradiation can be performed with both IR and UV lasers (see main text for details). The second window allows to decouple the laser from the chamber and perform power measurements while recording action spectra. In addition, a mirror is incorporated into the ToF pusher lens stack to allow for on-axis irradiation of the ions. This irradiation mode was not yet utilized in these experiments, in part due to the fact it is not possible to perform simultaneous pulse energy measurements. A detailed description of the parameters used for IR ion spectroscopy can be found in the main text.

(iv) Ejection and Detection

After irradiation T_2 will contain remaining precursor ions plus any photofragments that have been produced. These ions must be transferred to the pusher of the TOF in order to record a mass spectrum. Typical voltages used for this are shown in Figure S5 (green). Ejection of ions from T_2 to the TOF shares many features with transfer of ions from T_1 to Hex_2: the trapping potential on T_2 is removed, a positive potential applied to P_2 and a negative potential applied to L_3. There are some important differences. As L_3 also functions as the first acceleration electrode into the ToF, a larger negative potential is applied, corresponding to the value of *acceleration 1* in normal operation.

As with the discussed above, the time taken for ions to move from the T_2 to the pusher will depend on the mass-to-charge of the ions. This is measured for a variety of different m/z values in Figure S3, which shows ion count versus delay between ejection of ions from T_2 and activation of the pusher. The main complication in this case is that, following photoactivation, we may have many photofragments of different m/z. Figure S3 shows that it is not possible to record all masses simultaneously. Therefore, it is not possible to record the full mass spectrum with a single pusher delay – each pusher delay instead records a slice of the mass spectrum.

To overcome this problem, several different pusher delays are used within a single acquisition cycle of the mass spectrometer. So, for instance, if our trapping cycle lasts for 250 ms, we can set the acquisition time of the mass spectrometer 1 s and thus fit 4 cycles in this time. Each cycle will have a different pusher delay. As the ADC of the mass spectrometer sums all the mass spectra recorded during a single acquisition, the total mass spectrum can be recovered in this manner.

A further problem that can occur during detection is saturation of the detector. In normal operation mode, the ion density in any given push is relatively low, and the final mass spectrum is the sum of a large number of individual mass spectra with few ions. Here we have the opposite situation, many ions in a few mass spectra, as all the ions of a given m/z arrive at once to the pusher. We have found that typically we can accommodate around 10^3 ions of a given m/z in the trap before we begin to see saturation, and this is checked for every measurement. If required, we can limit the number of ions using the DRE lens of the Photo-Synapt.

Figure S3. Normalized intensity versus pusher delay.

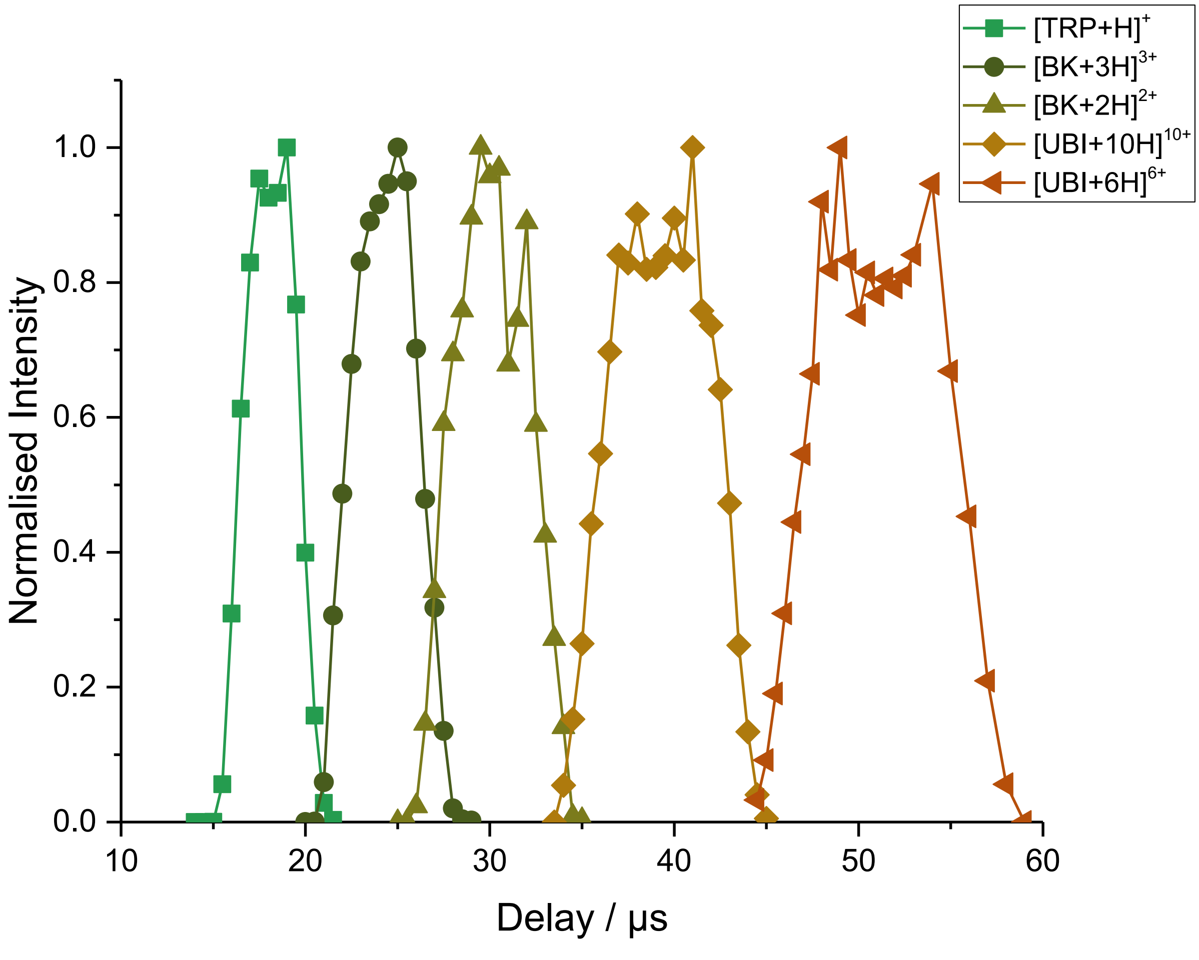

Figure S3. Normalized intensity vs pusher delay for ions of different m/z. Tryptophan (TRP) m/z 205, Bradykinin (BK) 3+ m/z = 354, 2+ m/z = 531. Ubiquitin (UBI) 10+ m/z = 859, 6+ m/z = 1431. Ions with a lower m/z reach the pusher sooner and thus a shorter delay between release of ions from the trap and triggering of the pusher is required.

Figure S4. Normalized ion count versus transfer time.

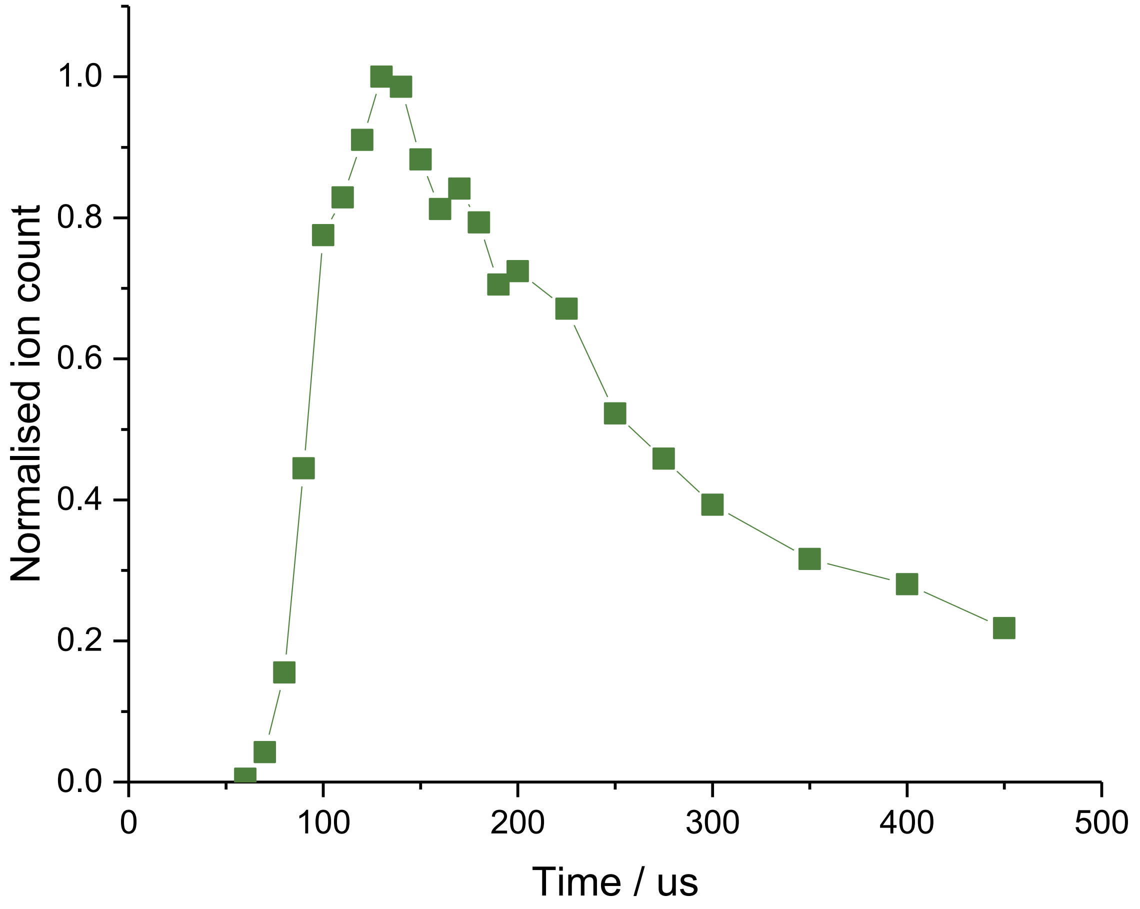

Figure S4. Normalized ion count versus transfer time for tryptophan 1+ ions. The time denotes how long the voltages are left in the transfer state, with P_1 = 5 V, L_2 = -1 V.

Figure S5. Schematic representation of voltages on the hexapole pin traps.

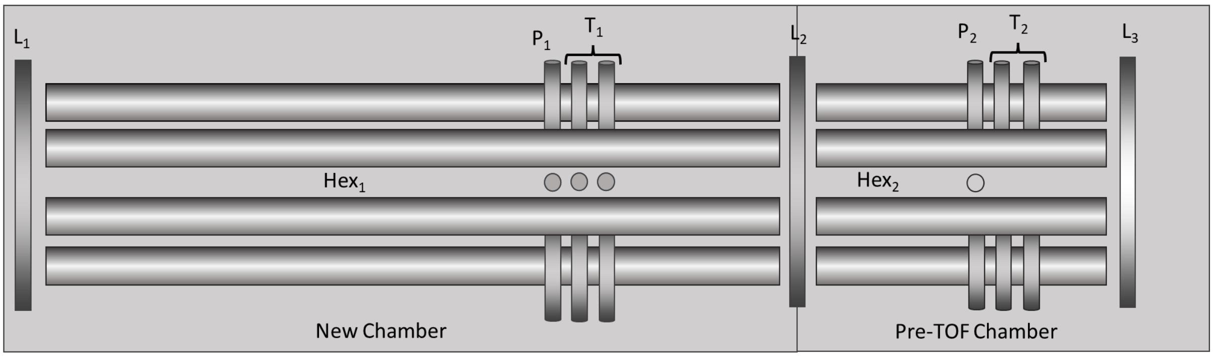

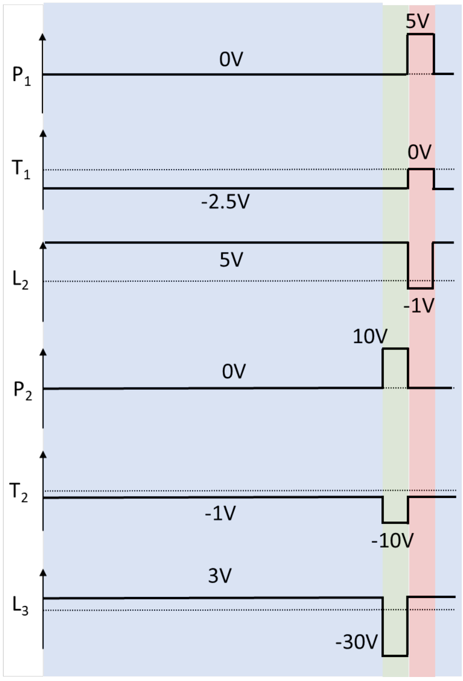

Lens	Trapping	Ejection	Transfer
L_1	0.5	0.5	0.5
P_1	0	0	5
T_1	-2.5	-2.5	0
L_2	5	5	-1
P_2	0	10	0
T_2	-1	-10	-1
L_3	3	-30	3

Figure S5. Schematic representation of the voltages on each of the ion optics during the trapping (blue) ejection (green) and transfer (ref) cycles.

Figure S6. Full MS of VEALYL.

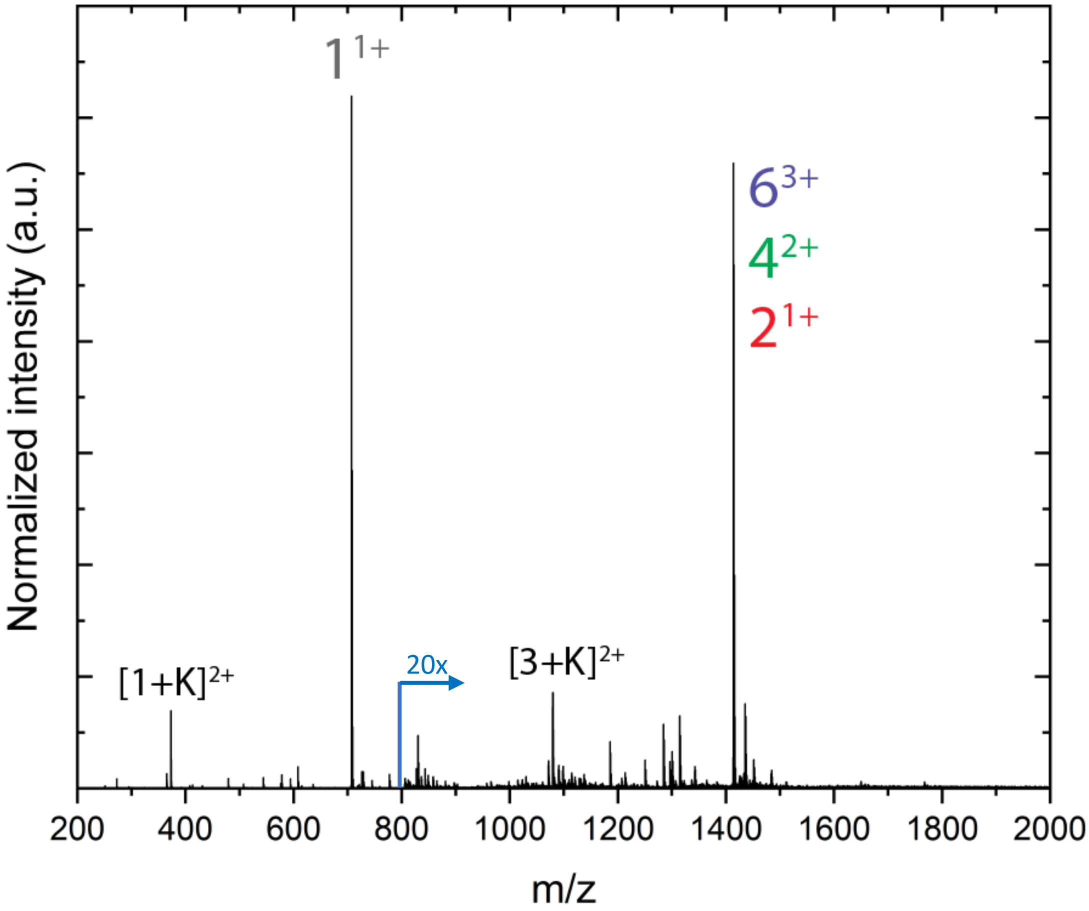

Figure S6. Full mass spectrum of VEALYL. For clarity all above 800 m/z have been multiplied by 20.

Figure S7. Fragmentation pathways for VEALYL using CID and IRMPD.

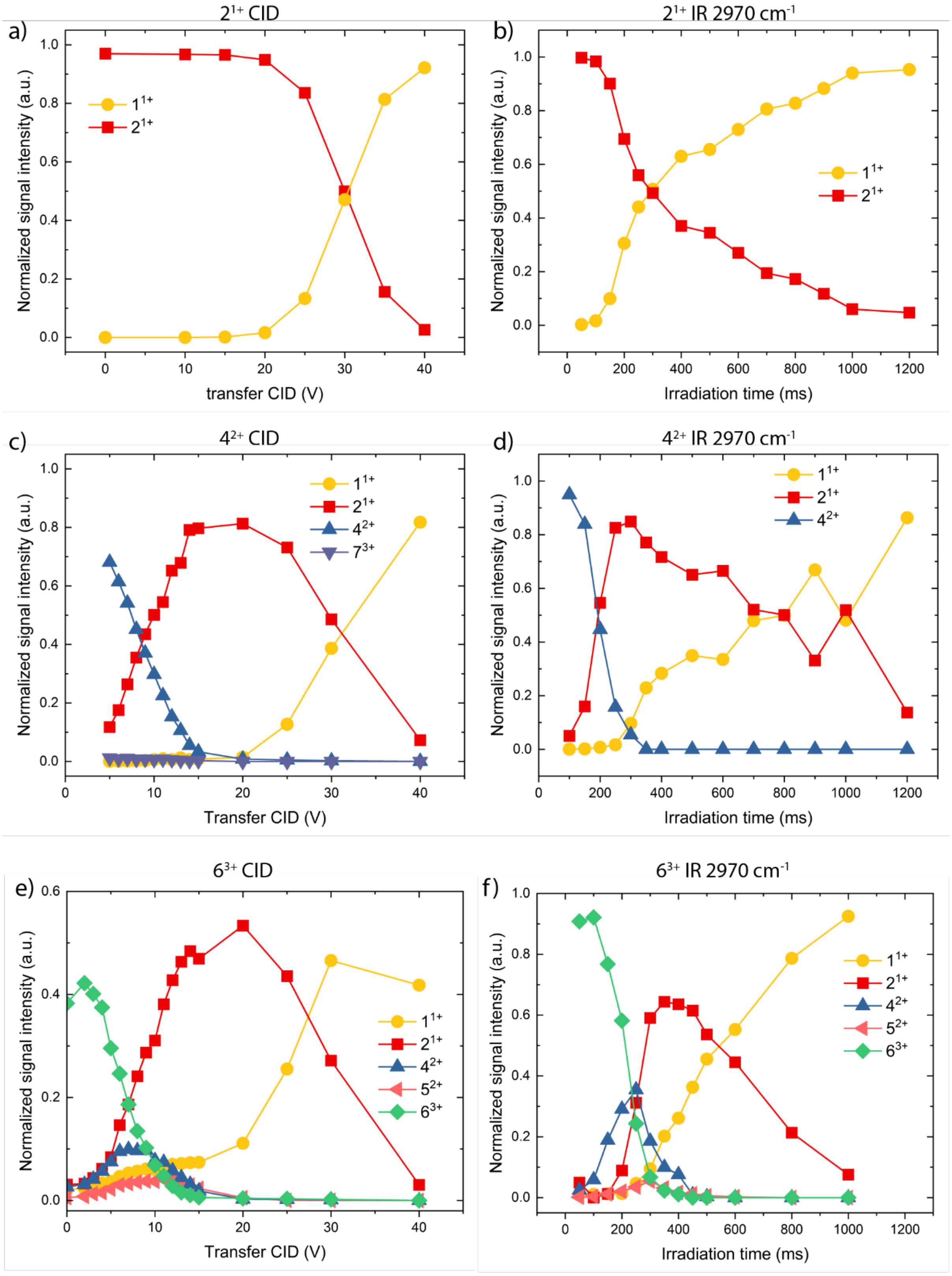

Figure S7. Fragmentation yield for several mass selected and ion mobility sliced species: 2^{1+}, 4^{2+} and 6^{3+} using CID (a, c and f, respectively), and using IR irradiation at different wavenumbers (b for 2^{1+}, d and e for 4^{2+} and g, h and I for 6^{3+}). The y-axes represent normalized intensities, and the x-axes either show the voltage (for CID) or the irradiation time in ms (for the IR measurements).

Section S2. Solving overlapping m/z species of VEALYL.

As stated in the main text, determining the IR photofragmentation yield, and thus plotting the IR spectrum, can be quite challenging when studying an aggregation process. Smaller oligomer-photofragments formed by photofragmentation of larger oligomers are stable in the trap and can absorb the IR radiation as well. This would result in an overlapping IR signature of the mass- and mobility-selected oligomer and the formed oligomer-photofragments, which often are lower-order oligomers that we just carefully removed either by the quadrupole or by ion mobility slicing. Therefore, it is essential to take only primary photofragments into account when determining the photofragmentation yield. Here, we explain this process for the 2^{1+}, 4^{2+} and 6^{3+} oligomers of VEALYL. To determine their fragmentation pathways, we performed some experiments where we monitored the fragments versus their irradiation time and, in separate experiments, the CID voltage, see Figure S7.

VEALYL 2^{1+}

The fragmentation pathway of the 2^{1+} is shown in Figure S7a (CID) and b (IRMPD): In both experiments it is clear that only 1^{1+} is formed. The precursor is m/z 1413, and its fragment m/z 707.

VEALYL 4^{2+}

It becomes more complex when we go to the 4^{2+}, which shares the same m/z value as the 2^{1+}. By slicing with ion mobility we ensure that only the 4^{2+} is present in the ion trap, but we see that its primary fragment is the 2^{1+}, see Figure S7c-d. Knowing the CID pathway of the 2^{1+}, we can see that the 4^{2+} fragments into the 2^{1+} at very low voltages, and is completely fragmented at 20V. At this point the 2^{1+} shows the same behavior as the 2^{1+} in Figure S7a, and we can conclude that using CID, the 1^{1+} fragments are secondary and don't arise from the 4^{2+}. Figure S7d shows again the fragments, but then using IRMPD at a resonance wavelength. The 4^{2+} is almost completely fragmented around 300 ms, at which the 1^{1+} only just starts to form in tiny amounts. This gives us the confidence that the same fragmentation pathway exists for IRMPD as it does for CID. The precursor and the fragment are therefore both at m/z 1413 which complicates the analysis. For this reason, we used the isotopic patterns as shown in Figure S8a (IR on resonance), and c. (off resonance).

The peaks are named with roman numerals from I to IX, starting from the monoisotopic peak. In Figure S8a the peaks I, V and IX have a distance of 1 between them, and are the isotopic peaks of the 2^{1+}, however, they also contain isotopic peaks of the 4^{2+}. In contrast, peaks III and VII only contain the 4^{2+}. In Table S1 we plotted the theoretical intensity for the isotopic peaks when either only 2^{1+} was present, or when only 4^{2+} was present. By taking the sum of two groups of peaks: One which contains both 2^{1+} and 4^{2+} ("A", peaks I, V and IX), and one which contains only the 4^{2+} ("B", peaks III and VII), and divide them to get the ratio, we can estimate the total intensity of the precursor.

We do this by first extracting the intensities of each of the individual peaks for the IR scan, and then sum them up as explained above. The 4^{2+} intensity is then calculated as the measured B peaks (III + VII), plus 1.13 x the same value of B. We then assume that there is 1.13 x B still in the group A peaks which should be attributed to 4^{2+}. In the same way we calculated the intensity of 2^{1+}: the measured value of A (I + V + IX), minus 1.13 x B. Although we end up with not purely measured quantities, we think this approaches the real picture the best. The main assumption we make is that the theoretical isotopic distribution is reflected in the measured spectra. An alternative, to use for example only the first and second isotopic peak to calculate the photofragmentation yield, has a relative good estimate of the 4^{2+}, but not at all of the 2^{1+}.

VEALYL 6^{3+}

The 6^{3+} can theoretically (by breaking non-covalent bonds) fragment into a large variety of different oligomers and charge states. When comparing the plots in Figure S7e-f, we can conclude that the 4^{2+} is a primary fragment of the 6^{3+}: in the IRMPD spectrum we don't observe the 5^{2+} as a possible intermediate fragment. The 2^{1+} seems to result from fragmentation of the 4^{2+} and cannot be taken into account as a primary fragment. For the analysis of the 6^{3+} we therefore need to distinguish between three oligomers with the same m/z. For this we named the peaks as shown in Figure S9a.

The same procedure was followed as for the 4^{2+}, in which we calculated the ratio between two groups of peaks (see Table S1), this time using the peaks that only contain the 6^{3+} (C), and that contain all three possible oligomers (A). The ratio between the two groups of peaks is 0.50, meaning that when we measure the total amount of signal of group C (6^{3+}), we will calculate the total signal of 6^{3+} as being C + 0.5 x C. The 4^{2+} is calculated in the same way as in the part above.

Figure S8. Theoretical and experimental isotopic distributions of VEALYL 4^{2+}.

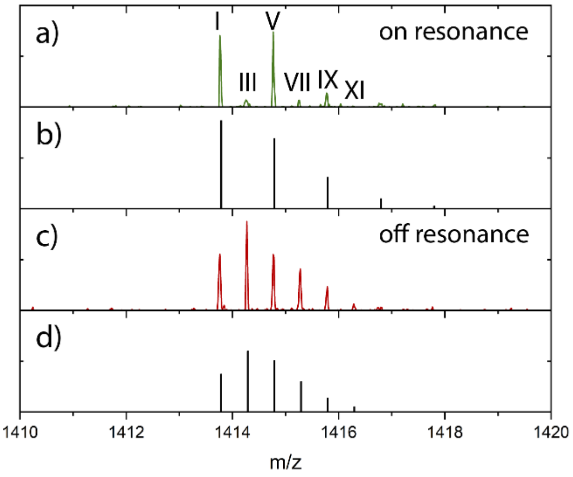

Figure S8. a) Mass spectrum measured at a resonance IR transition with b) the theoretical isotopic distribution of the 2^{1+} c) the mass spectrum without IR, and the theoretical isotopic distribution of the 4^{2+} oligomer.

Figure S9. Theoretical and experimental isotopic distributions of VEALYL 6^{3+}.

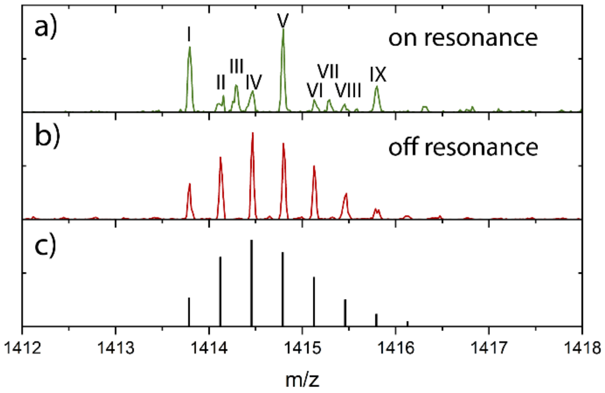

Figure S9. a) Mass spectrum of 6^{3+} at a resonance IR transition and b) off resonance, with c) the theoretical isotopic distribution of the 6^{3+} oligomer.

Table S1. Theoretical isotopic distributions of VEALYL 4^{2+} and 6^{3+}.

Peak	Containing	Only 2^{1+} (norm.)	Only 4^{2+} (norm.)	Only (6^{3+})
**I**	2^{1+}, 4^{2+}, 6^{3+}	0.46	0.20	0.08
**II**	6^{3+}			0.20
**III**	4^{2+}		0.31	
**IV**	6^{3+}			0.25
**V**	2^{1+}, 4^{2+}, 6^{3+}	0.37	0.26	0.21
**VI**	6^{3+}			0.14
**VII**	4^{2+}		0.16	
**VIII**	6^{3+}			0.08
**IX**	2^{1+}, 4^{2+}, 6^{3+}	0.17	0.07	0.04
A (I + V + IX)	2^{1+}, 4^{2+}, 6^{3+}		0.53	0.33
B (III + VII)	4^{2+}		0.47	
C (II + IV + VI + VIII)	6^{3+}			0.67
A/B			1.13	
A/C				0.50

Table S2. Theoretical isotopic distributions when only the 2^{1+} is present (second column), when only the 4^{2+} is present (third column), or when only the 6^{3+} is present (last column). The calculations in the bottom show the ratio between two groups of peaks.

Figure S10. Mass resolution after transmission and trapping

Trapping of the ions shifts the mass spectrum slightly (-0.15 at m/z 1413) and narrows the peaks, see Figure S10. The cause of the shift originates from the way we control the pusher plate via our own software and cannot be accounted for during the measurement. This means that a manual calibration is required after the measurement. The peak narrowing after trapping is a result from the configuration of the trap.

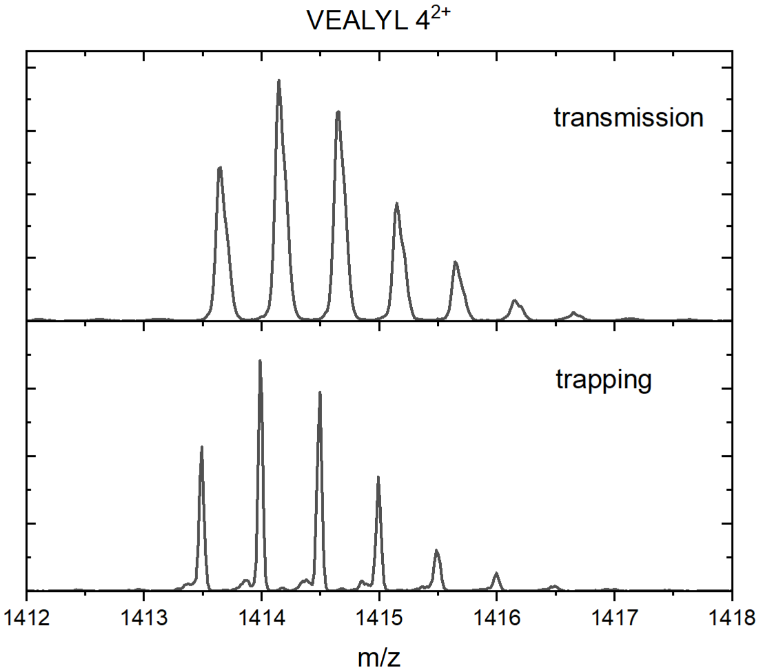

Figure S10. Mass spectra of the doubly charged tetramer of VEALYL. Top: in transmission mode, bottom: in trapping mode.

Figure S11. Raw IRMPD data for VEALYL oligomers.

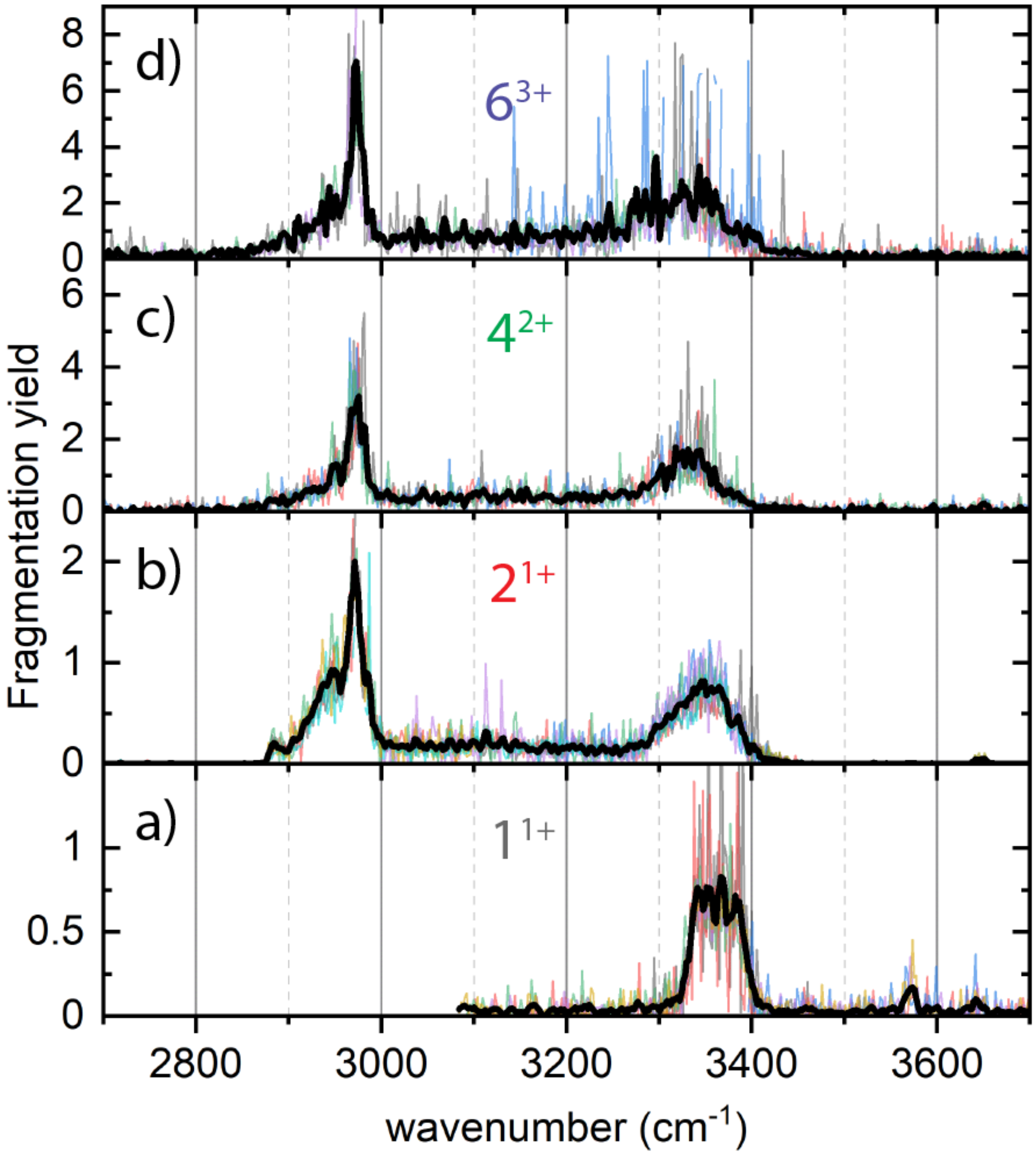

Figure S11. Raw yields (individual colors) and their smoothed averaged spectra (thick black line) for a) the 1^{1+}, b) 2^{1+}, c) 4^{2+} and d) the 6^{3+}.

Figure S12. Ion mobility analysis for glycan fragment m/z 657 of Transferrin (TF).

The m/z 657 fragment is formed by spraying the transferrin protein under harsh source condition, i.e. 2.1-2.3 kV capillary voltage, 90-130 V sampling cone. Subsequently, the pressure in T_2 was kept at 8.5e-7 mbar to minimize competition from collisional cooling and to maximize fragmentation. Figure S12 presents the extracted mass spectra of all different arrival time distributions of mass selected m/z 657. Peaks I, III and IV show different species than the mass of interest, peak II corresponds perfectly to the expected theoretical isotopic distribution and belongs to the fragment of interest.

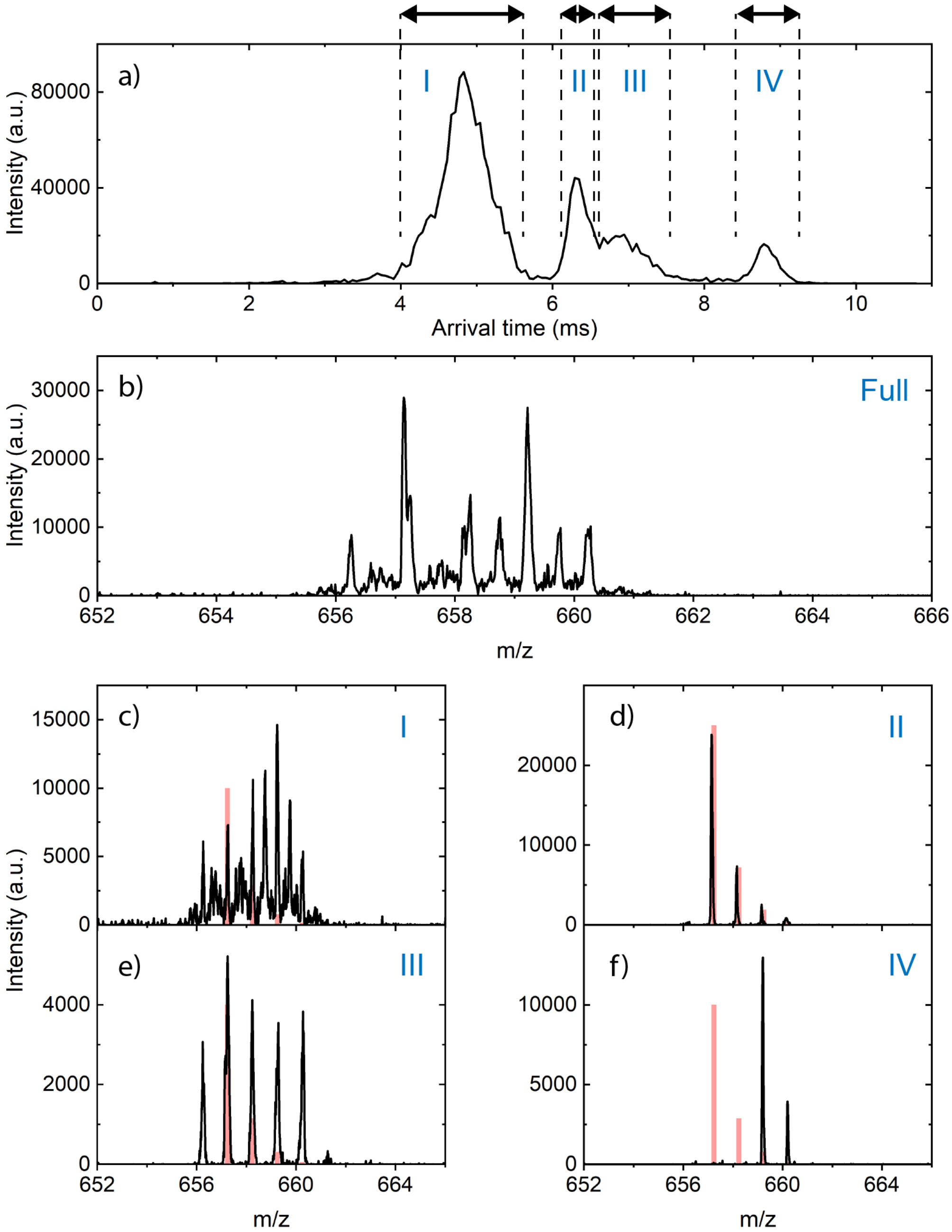

Figure S12. Glycan fragment m/z 657 of TF. a) Arrival time distribution of quadrupole selected m/z 657, peaks are indicated by roman numerals; b) full mass spectrum corresponding to a); c-f) corresponding mass spectra of each arrival time in a), indicated by the roman numerals. In red the theoretical isotopic distribution of the fragment of interest.

Figure S13. Ion mobility analysis for glycan fragment m/z 657 of hAGP.

Figure S13d-j shows the mass spectra belonging to each peak in the ion mobility spectrum of m/z 657 of hAGP. Peaks I, II and III are attributed to fragments created after quadrupole selection but before/during ion mobility. Peaks IV and VII are different species (VII) or background (IV), and peaks V and VI are the two different sialic acid conformers.

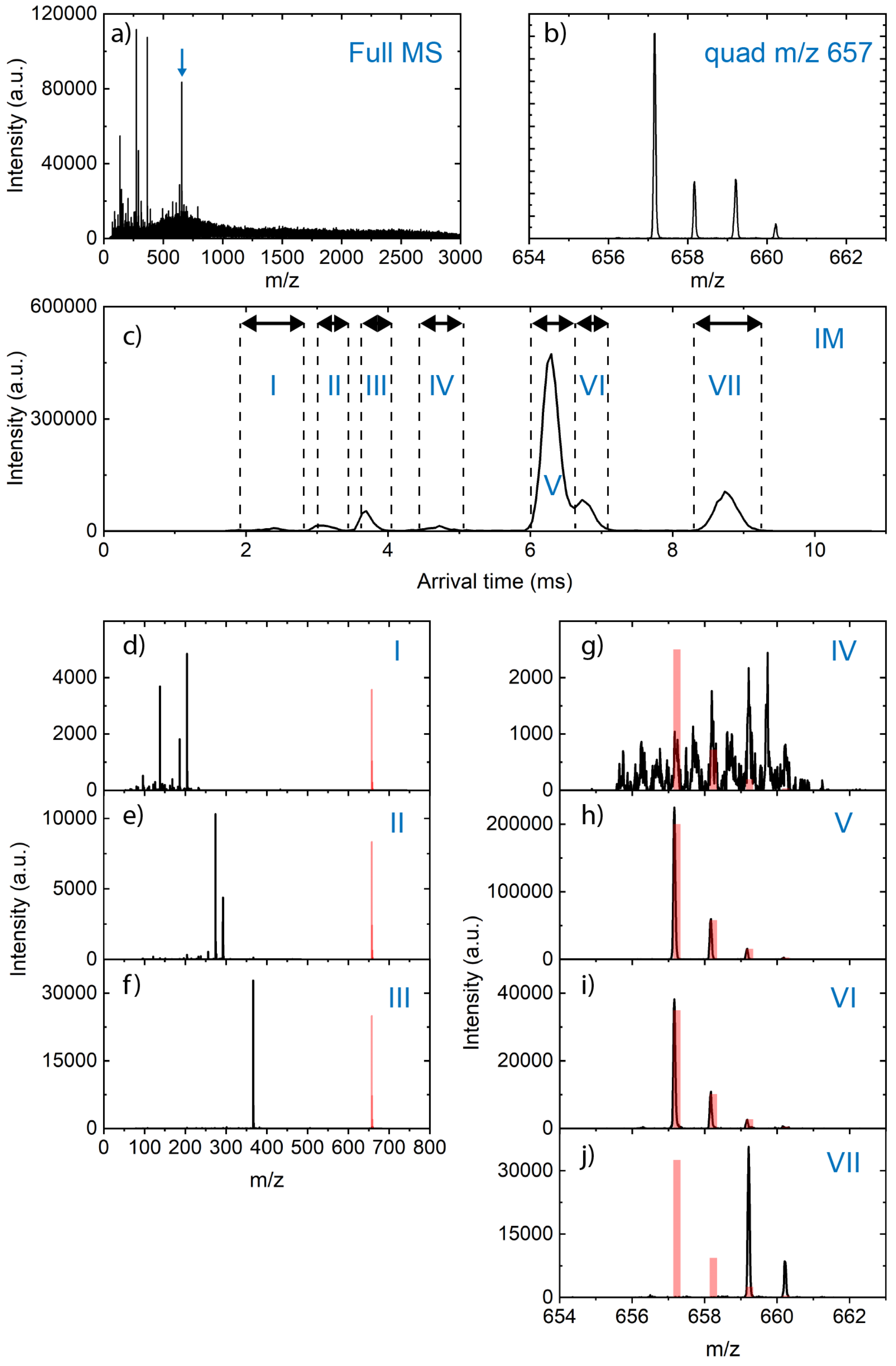

Figure S13. Glycan fragment m/z 657 of hAGP. a) Full MS; b) zoom-in of quadrupole selected m/z 657; c) arrival time distribution for m/z 657, peaks are indicated by roman numerals; d-j) corresponding mass spectra of each arrival time, indicated by the roman numerals. In red the theoretical isotopic distribution of the fragment of interest.

Figure S14. Raw IR data for m/z 657.

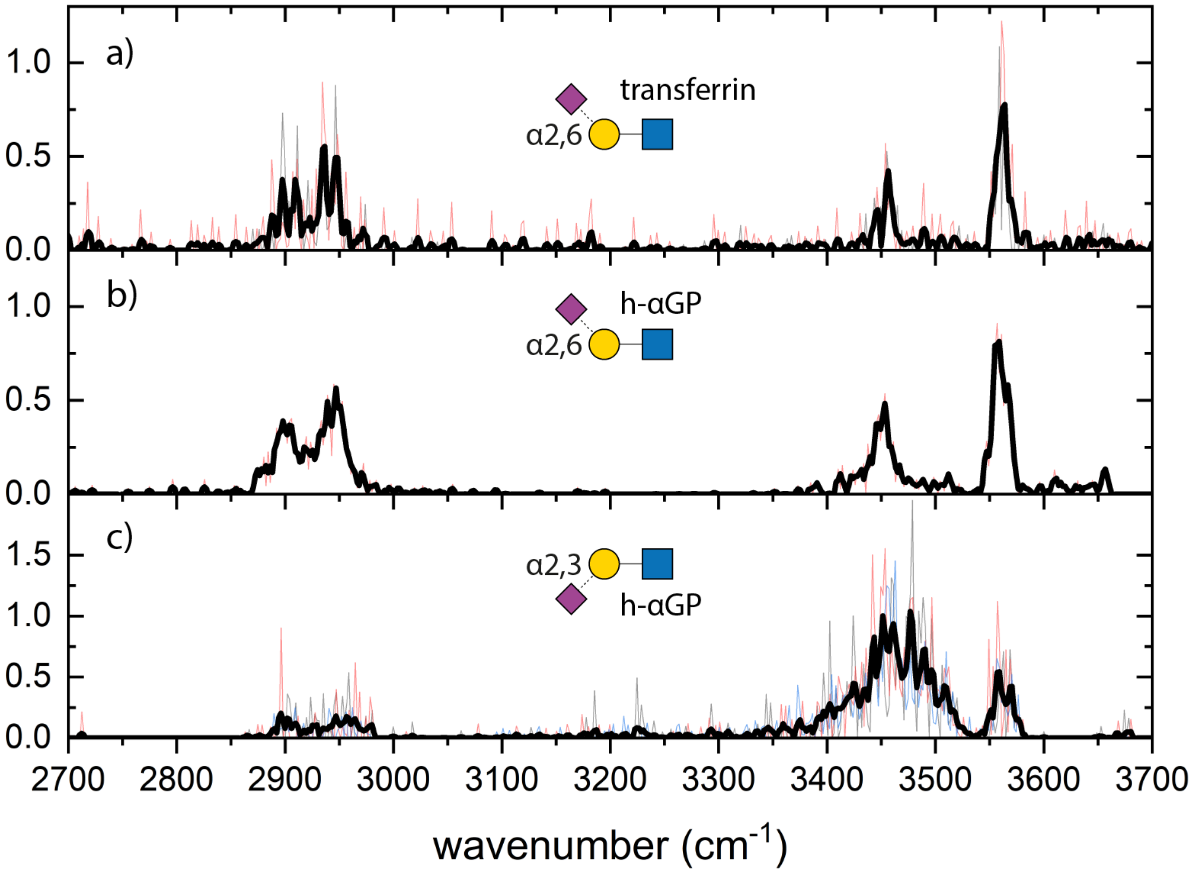

Figure S14. Raw IR data for ion mobility and mass selected glycan fragments with a) α2,6-linked sialic acid from transferrin N-glycans, b) α2,6-linked sialic acid of hAGP N-glycans and c) α2,3-linked sialic acid of hAGP N-glycans.
